# Evaluation of Various Solvent Extracts of *Tetrastigma leucostaphylum* (Dennst.) Alston Leaves, a Bangladeshi Traditional Medicine Used for the Treatment of Diarrhea

**DOI:** 10.3390/molecules25214994

**Published:** 2020-10-28

**Authors:** Sajib Rudra, Afroza Tahamina, Nazim Uddin Emon, Md. Adnan, Mohammad Shakil, Md. Helal Uddin Chowdhury, James W. Barlow, Mona S. Alwahibi, Mohamed Soliman Elshikh, Mohammad Omar Faruque, Shaikh Bokhtear Uddin

**Affiliations:** 1 Ethnobotany and Pharmacognosy Lab, Department of Botany, University of Chittagong, Chattogram 4331, Bangladesh; rudrasajibcu89@gmail.com (S.R.); tahaminasompurna@gmail.com (A.T.); shakilm115@gmail.com (M.S.); helaluddinchowdhurycu@gmail.com (M.H.U.C.); 2 Department of Public Health, School of Science and Technology, Bangladesh Open University, Gazipur 1705, Dhaka, Bangladesh; nazim7emon@gmail.com; 3 Department of Bio-Health Technology, College of Biomedical Science, Kangwon National University, Chuncheon 24341, Korea; mdadnan1991.pharma@gmail.com; 4 Department of Chemistry, Royal College Surgeons, Dublin D02YN77, Ireland; jambarlow@rcsi.ie; 5 Department of Botany and Microbiology, College of Science, King Saud University, Riyadh 11451, Saudi Arabia; malwahibi@ksu.edu.sa (M.S.A.); melshikh@ksu.edu.sa (M.S.E.)

**Keywords:** *Tetrastigma leucostaphylum*, ethnomedicinal plant, GC-MS, antidiarrheal, cytotoxicity, molecular docking and ADME/T

## Abstract

*Tetrastigma leucostaphylum* (TL) is an important ethnic medicine of Bangladesh used to treat diarrhea and dysentery. Hence, current study has been designed to characterize the antidiarrheal (in vivo) and cytotoxic (in vitro) effects of *T. leucostaphylum*. A crude extract was prepared with methanol (MTL) and further partitioned into *n*-hexane (NTL), dichloromethane (DTL), and *n*-butanol (BTL) fractions. Antidiarrheal activity was investigated using castor oil induced diarrhea, enteropooling, and gastrointestinal transit models, while cytotoxicity was evaluated using the brine shrimp lethality bioassay. In antidiarrheal experiments, all doses (100, 200, and 400 mg/kg) of the DTL extract significantly reduced diarrheal stool frequency, volume and weight of intestinal contents, and gastrointestinal motility in mice. Similarly, in the cytotoxicity assay, all extracts exhibited activity, with the DTL extract the most potent (LC50 67.23 μg/mL). GC-MS analysis of the DTL extract identified 10 compounds, which showed good binding affinity toward M3 muscarinic acetylcholine, 5-HT3, Gut inhibitory phosphodiesterase, DNA polymerase III subunit alpha, and UDP-*N*-acetylglucosamine-1 carboxyvinyltransferase enzyme targets upon molecular docking analysis. Although ADME/T analyses predicted the drug-likeness and likely safety upon consumption of these bioactive compounds, significant toxicity concerns are evident due to the presence of the known phytotoxin, 2,4-di-*tert*-butylphenol. In summary, *T. leucostaphylum* showed promising activity, helping to rationalize the ethnomedicinal use and importance of this plant, its safety profile following both acute and chronic exposure warrants further investigation.

## 1. Introduction

Infectious diseases are responsible for significant morbidity and mortality worldwide [1]. Developing countries are especially prone to the risk of these diseases, including diarrhea, influenza, and microbial infections [2]. Among these, diarrhea represents a sizeable burden, being regarded as the second most common cause of death in the world [3]. It has been reported that between 2 and 4 billion cases of diarrhea occur in poor countries each year, with children (age < 5) particularly susceptible. According to its duration, diarrhea is divided into three categories: acute (<2 weeks), intermittent (2–4 weeks) and persistent (over 4 weeks) [4]. The most common etiology of diarrhea is unhygienic living conditions, and subsequent gastrointestinal infection caused by parasites, viruses and other microorganisms may lead to a dysentery like chronic situation [5]. Such organisms are responsible for enteric infections, which manifest as one of two types of diarrheal disorder, namely a non-inflammatory or inflammatory presentation [6]. Many pathogens produce endogenous enterotoxins (e.g., cholera toxins, the heat-labile or stable enterotoxins of *Escherichia coli*), which actively invade cells and subsequently produce a non-inflammatory diarrheal syndrome. Other pathogens produce cytotoxins (e.g., *Shigella*, entero-invasive *E coli*, or *Clostridium difficile*) that disrupt cells or trigger cell proteins which stimulate the release of inflammatory mediators causing an inflammatory diarrheal syndrome [2]. Some diarrhea treatments address this pathophysiology in a relatively non-specific manner, often aiming to minimize bowel movement dysfunction and discomfort.

The World Health Organization (WHO) has recommended several approaches to alleviate the burden of diarrheal illness, of which a cornerstone is oral rehydration therapy. Of the established conventional antidiarrheal drugs, many are associated with adverse effects and contraindications [7]. In infectious diarrhea, a particular problem is the rapid resistance to antibiotics [8]. In addition to conventional medicines, traditional medicines are widely used by many cultures, but must be scientifically evaluated to assess their potential benefit or otherwise. Generally, traditional medicinal plants are rich in bioactive compounds, and plant derived medicines may provide complementary pharmacological actions in mitigating chronic disease through synergistic effects [9]. Hence, researchers are refocusing their attention on the evaluation of potential natural bioactive agents to treat diarrhea and other infectious diseases. Moreover, factors such as poor sanitation, lack of awareness, inadequate water and medical facilities have all been linked to transmission of such infections, especially in lower middle-income countries like Bangladesh [10]. Therefore, scientific validation of plants used in traditional medicine systems is important to reveal novel drugs that can provide viable alternative agents for the management of diarrhea.

One such species is *Tetrastigma leucostaphylum*, belonging to the family *Vitaceae*, and commonly known as Horina lata. The plant is a liana, which climbs using tendrils, and has palmately compound leaves. It is found in subtropical and tropical regions of Asia, Malaysia, and Australia, where it grows in primary rainforest, gallery and monsoon forests and in moister woodland. Species of the genus Tetrastigma are notable as the sole hosts of parasitic plants in the family Rafflesiaceae. In Bangladesh, *T. leucostaphylum* is found in the Chittagong Hill Tracts and Sylhet regions. Traditionally, the plant is very valuable. For example, the Chakma and Tripura indigenous communities use the leaves to treat diarrhea, stomach disorders and stomachache [11], while the juice expressed from its leaves is used by the Marma community for the treatment of diarrhea and a paste of the leaves is used to treat tumors (personal communication). The juice of the leaves has also been used alongside other, unknown ingredients to treat tetanus and paratyphoid by the Chakma indigenous community [11]. On the other hand, the root of the plant is used to treat fever, gout and oedema by both Marma and Pankhua indigenous communities. In spite of such widespread use, the potential of *T. leucostaphylum* as an anti-diarrheal and cytotoxic agent has not yet been scientifically evaluated. In fact, the only report on bioactivity of the species is an evaluation of the acaricidal activity of the petroleum ether extract of its leaves [12]. Therefore, we aimed to evaluate both the antidiarrheal effects (in vivo) and the cytotoxicity (in vitro) of various solvent extracts of *T. leucostaphylum*. In addition, bioactive constituents of *T. leucostaphylum* were identified using gas chromatography-mass spectroscopy (GC-MS), while in silico molecular docking analysis was performed in order to identify potential lead compounds and obtain insights into their potential mechanism(s) of action in the management of diarrheal diseases.

## 2. Results

### 2.1. GC-MS Analysis

**Figure 1:**
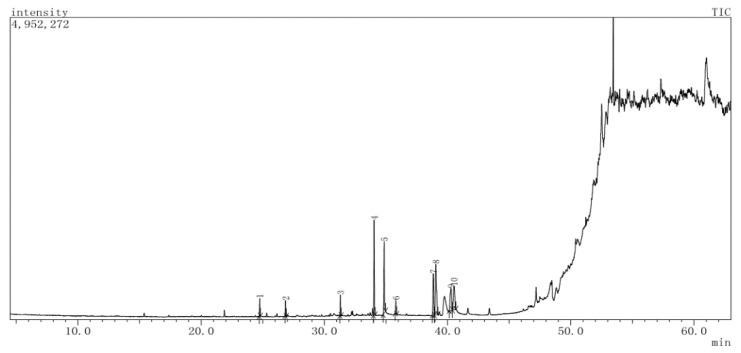
Total ion chromatogram (TIC) of dichloromethane (DTL) extract of *T. leucostaphylum*.

GC-MS analysis of the dichloromethane extract (DTL) revealed 10 compounds having retention times of between 24.75 (2,4-di-*tert*-butylphenol) and 40.51 (linolenic acid) min. The compounds, along with their chemical composition are listed in Table 1, whereas the total ionic chromatogram (TIC) is displayed in Figure 1. The highest peak area was exhibited by methyl elaidolinolenate (20.75%), followed by palmitic acid (16.62%), methyl palmitate (16.41%), linolenic acid (12.63%), methyl lineoleate (12.14%), linoleic acid (10.60%), nonadecene (3.20%), 2,4-di-*tert*-butylphenol (2.79%), behenic alcohol (2.66%), and pentadecene (2.21%).

### 2.2. In Vivo Antidiarrheal Activity

#### 2.2.1. Acute Oral Toxicity Test

For all extracts, a dose range of 50 to 3000 mg/kg did not result in any unusual signs of toxicity or behavioral abnormalities (sedation and over-excitation) during the acute toxicity test. In addition, mortality and other physical changes (allergic reaction and weight loss) were not observed for 72 h after oral treatment of all extracts, which suggested that the extracts used in this study have no toxic effects up to 3000 mg/kg (data not shown).

#### 2.2.2. Castor Oil Induced Diarrhea

The antidiarrheal potential of the crude extract (MTL) and further fractions (NTL, DTL, and BTL) of *T. leucostaphylum* on castor oil-induced diarrhea is shown in Table 2. In this test, all extracts demonstrated significant inhibition of diarrhea in a dose dependent fashion. Among all extracts, DTL was found to be most potent, with the overall effects of DTL higher than those of the reference drug loperamide, considering all parameters. Maximum inhibition of defecation by DTL was observed at 400 mg/kg (88.01%), followed by 200 mg/kg (76.15%) and 100 mg/kg (68.06%), while loperamide caused 75.44% inhibition at a dose of 3 mg/kg. Other fractions, including BTL, NTL, and MTL caused considerable delays in initiation of diarrhea at a dose of 400 mg/kg. Suppression of diarrhea at this dose was 71.09% for NTL, 51.87% for BTL, and 46.96% for MTL, respectively.

#### 2.2.3. Effects on Castor Oil Induced Enteropooling

In this test, DTL exhibited a remarkable reduction in both the mean weight and volume of intestinal contents (MWSIC and MVSIC) at all tested doses as compared to control (Table 3). Moreover, DTL at a dose of 400 mg/kg manifested noteworthy inhibition of weight (93.75%) and volume (91.17%) of intestinal contents in comparison to the reference drug loperamide (75% in MWSIC and 76.5% in MVSIC). The effects of other doses (100 and 200 mg/kg) of DTL were moderate, but significant compared to control and reference drug. In addition, NTL, BTL, and MTL revealed dose dependent reductions of MWSIC and MVSIC, while the percent inhibition of weight and volume of intestinal contents for all extracts was promising, and also comparable to the reference drug.

#### 2.2.4. Gastrointestinal Motility Test

The effects of different extracts of *T. leucostaphylum* on intestinal transit are summarized in Table 4. In this test, compared to other extracts, DTL impressively delayed intestinal transit of charcoal at every dose. The results revealed that the gastrointestinal movement of charcoal was significantly inhibited by 84.28%, 72.17%, and 66.46% at 400, 200 and 100 mg/kg doses respectively, while the standard drug (loperamide 3 mg/kg) caused a 75.19% anti-motility (charcoal inhibition) response. Furthermore, DTL at 400 mg/kg reduced the peristalsis index (14.76%) while for loperamide (3 mg/kg) it was 30.28%.

#### 2.2.5. Antidiarrheal Index

The in vivo antidiarrheal index (ADI) is shown in Table 5. The ADI was dose dependently increased at all doses of extracts. Moreover, the highest ADI was achieved in the case of DTL (164.69%), followed by NTL (140.44%), BTL (117.27%), and MTL (82.22%), at a dosage of 400 mg/kg.

### 2.3. Cytotoxicity Assay

**Figure 2:**
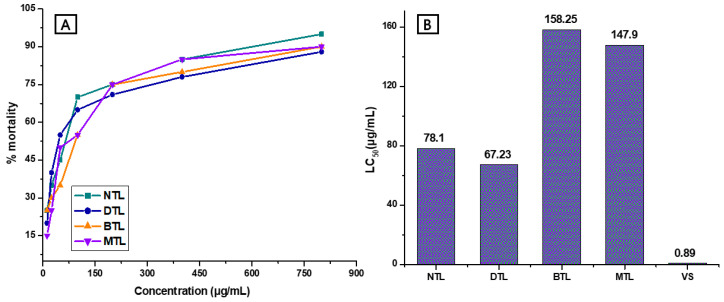
Cytotoxic effects of the methanol extract of the *T. leucostaphylum* leaves and its derived fractions against brine shrimp naupli. (**A**): Relation between extract concentration and percentage mortality; (**B**): LC50 values for cytotoxicity assay. NTL = *n*-hexane fraction of *T. leucostaphylum*; DTL = dichloromethane fraction of *T. leucostaphylum*; BTL = *n*-butanol fraction of *T. leucostaphylum*; MTL = methanol extract of *T. leucostaphylum*; vs. = vincristine sulfate.

The cytotoxicity results for MTL and its related fractions BTL, DTL, NTL, and vincristine sulfate (reference drug) are shown in Figure 2A,B, expressed as the mortality rate of brine shrimp naupli in the brine shrimp lethality bioassay. The highest LC50 (median lethal concentration of the test organisms) value was estimated as 158.25 μg/mL for BTL, followed by 147.9 μg/mL for MTL, 78.10 μg/mL for NTL and 67.23 μg/mL for DTL, respectively, while the LC50 values for vincristine sulfate was determined to be 0.89 μg/mL.

### 2.4. In Silico Studies

#### 2.4.1. Molecular Docking Studies

**Figure 3:**
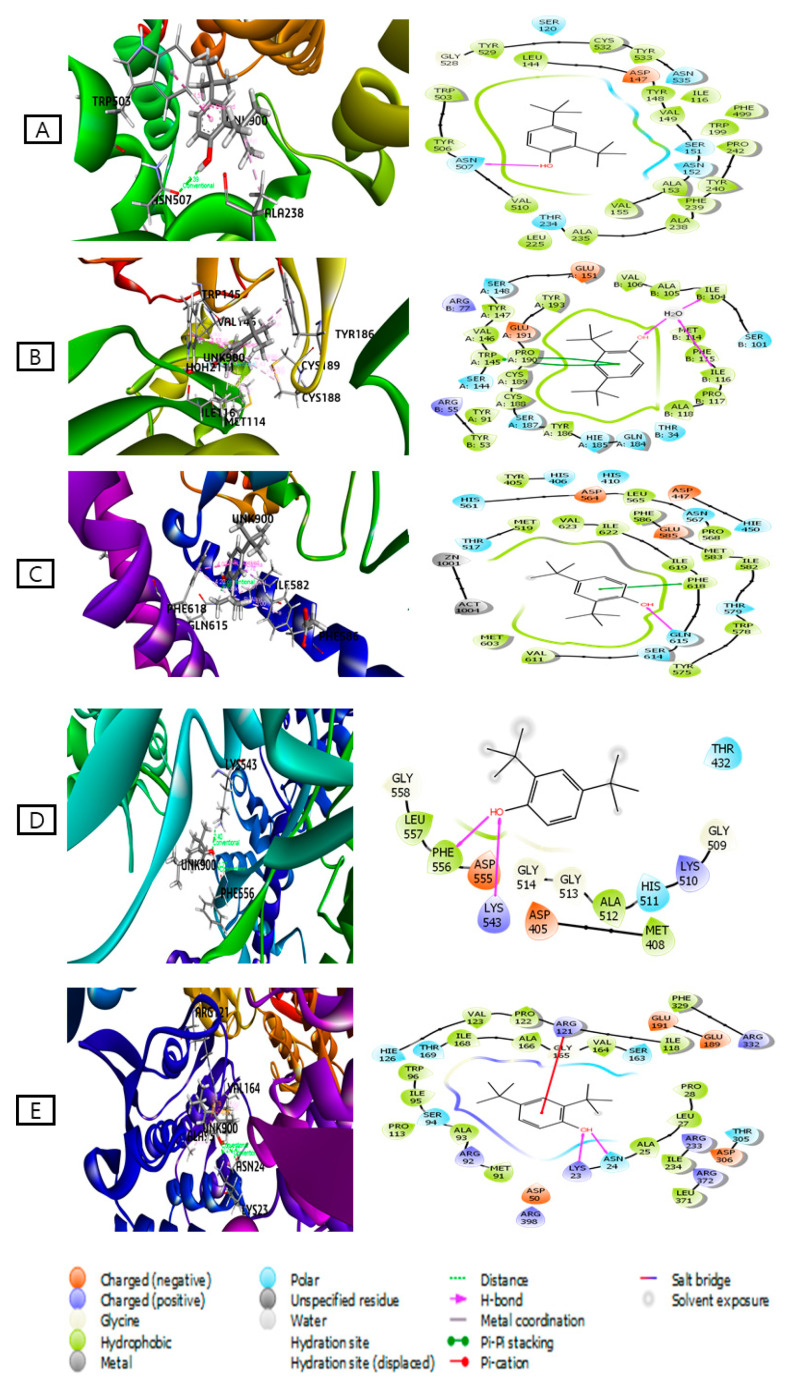
Best mode of 3D and 2D interaction of 2,4-di-*tert*-butylphenol with respective target proteins: (**A**) M3 muscarinic acetylcholine receptor (PDB ID: 4U14); (**B**) 5-HT3 receptor (PDB ID: 5AIN); (**C**) Gut inhibitory Phosphodiesterase receptor (PDB ID: 5LAQ); (**D**) DNA polymerase III subunit alpha (PDB ID: 4JOM); (**E**) UDP-*N*-acetylglucosamine-1-carboxyvinyltransferase (PDB ID: 4R7U). Variable colors reveal the presence of variant residue type: Orange-Acidic (Asp, Glu); Green-Hydrophobic (Ala, Val, Ile, Leu, Tyr, Phe, Trp, Met, Cys, Pro); Purple-Basic (Lys, Arg); Sky blue-polar (Ser, Thr, Gln, Asn, His, Hie); Light gray-other (Gly, Water) and Dark gray-metal atoms. Several interactions with the target receptor are drawn with lines between ligand atoms and protein residues: Solid pink: H-bonds to the protein backbones, Green: Pi-Pi stacking interactions, Red: Pi-cation interactions. Ligands solvent exposure is traced with gray spheres. The line exhibited around the ligand is noted as “Protein Pocket’’ and is marked with the color of adjacent protein residues. The disintegration of the lines displayed the onset pocket of protein.

All 10 identified compounds within the dichloromethane extract (DTL) were docked against five major receptors (M3 muscarinic acetylcholine receptor, PDB: 4U14; 5-HT3 receptor, PDB: 5AIN; Gut inhibitory Phosphodiesterase receptor, PDB: 5LAQ; DNA polymerase III subunit alpha, PDB: 4JOM; and UDP-*N*-acetylglucosamine-1 carboxyvinyltransferase, PDB: 4R7U) to determine their possible antidiarrheal potential (Table 6). From the docking analysis, it can be seen that 2,4-di-*tert*-butylphenol demonstrated the best docking score against all the receptors (Figure 3). The docking scores of this compound were higher than the standard drug loperamide. The ranking order based on the docking scores of antidiarrheal effects is as follows: 2,4-di-*tert*-butylphenol > behenic alcohol > methyl elaidolinolenate > linoleic acid > methyl lineoleate > linolenic acid > methyl palmitate > palmitic acid > nonadecene > pentadecene. The docking figures of other compounds are shown in Figures S1–S5. As 2,4-di-*tert*-butylphenol showed the highest binding affinity against all receptors, further binding interactions to all receptors are presented in Table 7.

#### 2.4.2. ADME Analysis

From Table 8, it can be observed that all compounds except 2,4-di-*tert*-butylphenol violated one parameter among Lipinski’s rule of five. In contrast, 2,4-di-*tert*-butylphenol met all Lipinski’s conditions, which indicate that this compound can be considered to be bioavailable.

## 3. Discussion

Infectious diarrhea remains a common problem throughout the world [13]. To address this, different countries utilize various remedial approaches; while developed countries utilize both rehydration strategies and symptomatic treatments, developing countries heavily depend upon oral rehydration solutions (ORS), with a lack of cheap and useful other medications [14]. However, tribal communities prefer traditional medicines as their primary health care system owing to low cost, affordability, and accessibility [15]. As ethnic medicines, although widely practiced, are largely based on empirical evidence, it is necessary to scientifically evaluate the pharmacological potential of traditional plants [16]. For example, a recent Ethiopian study evaluated several traditional medicines, including *Calpurnia aurea*, *Corton marcostachyus*, and *Echinopia kebercho* as anti-diarrheal agents [17]. To evaluate the intrinsic anti-diarrheal value of a plant, various efficient extraction techniques such as soxhlet extraction, ultrasonic-assisted extraction, subcritical water extraction, supercritical fluid extraction, and maceration with suitable solvents are prerequisites. Moreover, plant bioactives represent diverse chemical characteristics and polarities that are not all readily soluble in a particular solvent [18]. In this regard, solvent extraction followed by successive fractionation can be used to obtain semi-purified extracts, which further help to identify potential lead compounds [19]. Thus, to probe the potential therapeutic value of *T. leucostaphylum*, a crude extract was initially prepared in methanol, a solvent with strong extraction power. We subsequently adopted a successive solvent extraction approach, by partitioning the crude extract into *n*-hexane (NTL), dichloromethane (DTL), and *n*-butanol (BTL) fractions, which were subjected to cytotoxicity and antidiarrheal analysis for the first time.

Traditional management of diarrheal conditions with *T. leucostaphyllum* is very popular among tribal communities; hence, to expose the ethnomedicinal value of this plant, we determined the antidiarrheal activity of *T. leucostaphyllum* (crude and fractionated) extracts as well as its mechanism(s) of action in various diarrheal models. Diarrhea usually occurs whenever there is fluid imbalance in the gastrointestinal tract and/or if motility of smooth bowel muscles becomes disrupted [20]. The natural laxative castor oil is a commonly employed diarrheal stimulator. It generates an irritant laxative effect through release of its active metabolite, ricinoleic acid, which promotes peristaltic activity in the upper part of the small intestine, leading to alterations in electrolyte permeability of the intestinal mucosa [21]. In addition, ricinoleic acid induces localized inflammation and irritation of the intestinal mucus which gradually enhances gastrointestinal motility by inhibiting water and electrolyte absorption from the intestine [22].

In our study, administration of a high dose of castor oil (0.5 mL) to mice produced diarrhea, and all the tested doses (100–400 mg/kg) of *T. leucostaphylum* (crude and fractionated) extracts caused significant inhibition of diarrhea, evidenced by a dose-dependent reduction in fecal rate. Among all fractions, the highest inhibitory effect was observed at 400 mg/kg of DTL extract, which strongly delayed diarrheal development and decreased both wet and overall fecal production and weight. This showed that a relatively high dose is required to elicit a significant antidiarrheal response, which is in line with previously published reports on different plant species [23].

The anti-enteropooling efficacy of *T. leucostaphylum* (crude and fractionated) extracts was explored to further investigate mechanistic aspects of activity. The presence of castor oil in the intestine stimulates the release of various pro-inflammatory mediators, such as prostaglandins, nitric oxide and histamine, which induce gastrointestinal motility [24]. In our experiments, compared to other fractions, the DTL extract clearly and dose-dependently repressed castor oil induced enteropooling into the small intestine. The DTL extract (100–400 mg/kg) caused a significant reduction in the volume and weight of the intestinal contents. At the higher dose of 400 mg/kg, DTL exerted the greatest inhibitory effect on both volume and weight of intestinal content, higher than the standard drug loperamide. This response indicates that *T. leucostaphylum* reduces the speed of intraluminal fluid, which may be due to inhibition of castor oil induced prostaglandin biosynthesis.

To further explore the effect of *T. leucostaphylum* (crude and fractionated) extracts on intestinal hypermotility, we assessed gastrointestinal motility using a charcoal meal tracer. We observed that all extracts acted as an effective suppressant of the passage of the charcoal meal through the entire intestine. This lowering of peristalsis supports that the extracts have antimotility properties. In addition, the peristaltic index exhibited by DTL at 400 mg/kg was comparable to the standard drug loperamide. Thus, *T. leucostaphylum* decreases hypermotility and increases transit time via suppression of intestinal muscle spasm, and may extend the duration of absorption [25].

The ADI value is an indication of the efficiency of an extract in the management of diarrhea [26]. In our study, the ADI value increased with increasing dose of the extracts, suggesting a dose response relationship with this parameter. Among all fractions, the DTL extract showed the highest ADI, suggesting that this extract has greater potential than other solvent fractions. Overall, the data obtained from our animal models suggests that *T. leucostaphylum* may be a good candidate for diarrhea of manifold causes, including due to infectious agents.

To evaluate the possible cytotoxic properties of the *T. leucostaphyllum* (crude and fractioned) extracts, we conducted a brine shrimp lethality bioassay (BSLB). This method provides a preliminary insight of anticancer and pesticidal efficacy of bioactive compounds present in the plant extracts [27,28]. Our results showed a concentration dependent pattern of cytotoxicity, with all extracts expressing slight to strong toxicity, and maximum mortality observed at a dose of 800 µg/L. In addition, the recorded lethal concentration (LC50) indicated that the DTL extract was relatively more toxic at lower concentration compared to other extracts. In the case of the brine shrimp lethality bioassay, cytotoxic activity is considered to be weak with an LC50 between 500 and 1000 μg/mL, moderate between 100 and 500 μg/mL, and strong between 0 and 100 μg/mL [29]. While the DTL extract was the most potent, reasonable cytotoxic actions were also noted for other extracts. Presumably, the potential toxic compounds are secondary metabolites present in the extracts and exhibit ovicidal and larvicidal properties [30]. Interestingly, medicinal plant extracts found to be toxic in the BSLB are likely to be candidates for further biological research [31].

As we know, plants are prolific sources of secondary metabolites [32] which have various roles, including as natural defense mechanisms against infective agents [33]. A preliminary phytochemical investigation of *T. leucostaphylum* indicated various such phytoconstituents, including steroids, alkaloids, tannins, and flavonoids (data not shown). The literature reports that these metabolites can all can exert antidiarrheal effects [34], and tannins and flavonoids are particularly known to promote reabsorption of intestinal fluids and electrolytes [35]. As the DTL extract showed the most promising antidiarrheal activities, we subjected it to GC-MS analysis, which identified the presence of ten bioactive compounds, all of which are noted in the literature. Of these, 2,4-di-*tert*-butylphenol is particularly notable, having been reported as a common toxic secondary metabolite which possesses antioxidant, anti-inflammatory, cytotoxic, insecticidal, nematicidal, antibacterial, antiviral, antifungal and phytotoxic properties [36].

Computational study viz. molecular docking is widely used for the prediction of ligand-target interactions and to get better insights into the biological activity of natural products [37]. It also gives further insights about the possible mechanism(s) of action and binding modes inside the binding pocket of enzymes [38]. To obtain a better insight into the observed antidiarrheal effects of the DTL plant extract, the ten identified metabolites were subjected to docking analysis. Compounds were docked against five target proteins, i.e., the M3 muscarinic acetylcholine receptor (PDB: 4U14), 5-HT3 receptor (PDB: 5AIN), gut inhibitory phosphodiesterase receptor (PDB ID: 5LAQ), DNA polymerase III subunit alpha (PDB ID: 4JOM), and UDP-N acetylglucosamine-1-carboxyvinyltransferase (PDB ID: 4R7U). Negative and low binding energies demonstrates strong favorable interactions of the ligand-receptor complex [39]. The docking study showed that 2,4-di-*tert*-butylphenol had strong binding affinities with the target proteins. From these results, the identified compounds might in some measure be accountable for the antidiarrheal effect of the plant extract by establishing several interactions with target proteins.

All compounds were further studied using the online prediction program ADME analysis, which evaluated drug-likeness, pharmacokinetics, and physiochemical characteristics of the compounds based on Lipinski’s rule of five [3]. Compounds with a molecular weight < 500 amu, < 10 Hydrogen bond acceptors, < 5 Hydrogen bond donors, and a lipophilicity value (LogP) ≤ 5 have high permeability [40], and good absorption and bioavailability characteristics [41]. Almost all 10 compounds had orally active drug-likeness properties according to Lipinski’s rule. Although 2,4-di-*tert*-butylphenol complied with Lipinski’s rule, indicating that it would have good oral bioavailability and could be considered as a possible lead compound, given the toxicity concerns associated with this compound, further study is warranted in this regard.

## 4. Materials and Methods

### 4.1. Collection and Identification

Leaves of *Tetrastigma leucostaphylum* were collected from Beganasori Village Common Forest (VCF), Rangamati (22°37′59.99″ N 92°11′60.00″ E), Bangladesh, during November 2017. Enhanced care was taken to avoid contamination and to only obtain only healthy, fresh specimens. A voucher specimen (accession no: CTGUH SR7912) was authenticated by Dr. Shaikh Bokhtear Uddin, Professor, Department of Botany, University of Chittagong-4331, Bangladesh, and deposited in the Herbarium of Chittagong University.

### 4.2. Extraction and Fractionation

The fresh leaves (with stems) were washed, dried, and pulverized into powder at room temperature using an electric grinder. The fine powder (585 g) was soaked in 2.5 L of methanol (Merck, Darmstadt, Germany) for 15 days, with regular shaking and stirring. The resulting extract was poured through a cotton plug and further filtered through Whatman No.1 paper, before evaporation on a rotary evaporator at 45 °C to afford the crude methanol extract of *T. leucostaphylum* (MTL: 95.78 g). Same extraction process was repeated three times and all obtained crude extracts were combined before further extraction with solvents. Following the customized protocol of kupchan partitioning [42], the crude methanol extract (10 g) was further partitioned to yield the following fractions; *n*-hexane (NTL), dichloromethane (DTL), and *n*-butanol (BTL) fractions.

### 4.3. Gas Chromatography/Mass Spectrometry (GC–MS) Analyses

GC-MS analysis of *T. leucostaphylum* (DTL extract) was analyzed through a Shimadzu gas chromatography-mass spectrometer (Shimadzu GC–MS QP2010PLUS, Shimadzu Corporation, nakagyo-ku, Japan ) and a DB-5MS capillary column (30 m × 0.25 μm × 0.25 mm). Detail protocol was described in our previous study [10].

### 4.4. Drugs and Chemicals

Vincristine sulfate was supplied by Beacon Pharmaceuticals Ltd., Dhaka, Bangladesh; 0.9% sodium chloride solution was obtained from Orion Pharmaceuticals Ltd., Dhaka, Bangladesh. DMSO (dimethyl sulfoxide) and Tween 80 were supplied by Sigma-Aldrich Co., St. Louis, MO, USA. Castor Oil was purchased from WELL Heath Care, Madrid, Spain. 10% charcoal in 5% gum acacia and loperamide were obtained from Square Pharmaceuticals Ltd., Dhaka, Bangladesh.

### 4.5. In Vivo Antidiarrheal Activity

#### 4.5.1. Experimental Animals

Swiss Albino mice having a body weight of around 25–30 g was collected from the Jahangir Nagar University (JU), Savar, Dhaka, Bangladesh. The collected mice were provided with standard laboratory feed and distilled water ad libitum, and acclimatized to day and night airflow patterns. The experimental protocol was approved by the Bio-safety, Bio-security and Ethical committee of Jahangirnagar University [BBECJU/M2018(3)1], Savar, Bangladesh. The experimental mice were managed according to the “Guide for the Care and use of Laboratory Animals, 8th ed” USA [43]. All studies were performed (9.00 a.m. and 5.00 p.m.) in a restricted and infinitely adjustable area (remote and noiseless ambiance).

#### 4.5.2. Acute Oral Toxicity Test

Acute oral toxicity was tested according to the OECD 425 guideline [44]. For the test, Swiss albino mice (*n* = 6) were divided into control and test groups. Each animal was administered 1% Tween 80 (control) or a single oral dose (50, 100, 400, 1000, 2000, or 3000 mg/kg body weight) of plant extract. Prior to the experiment, mice were fasted overnight and food was delayed for 4 h after administration of the extract. The treated mice were observed individually for any unexpected response and mortality over the next 72 h.

#### 4.5.3. Experimental Design

For the antidiarrheal experiments, thirty mice (both sexes) were separated into five groups (negative control, positive control or one of three observational groups), with six mice in each group. In all tests, loperamide (3 mg/kg, b.w, p.o) was used as a standard drug and 1% Tween 80 (10 mL/kg, p.o) was used as control. Different doses (100, 200 and 400 mg/kg, b.w, p.o) of crude extract were administered from specific fractions (groups III, IV and V). The standard drug was administered 15 min before and the crude extract (100, 200 and 400 mg/kg, b.w, p.o) or control 30 min before the experiments.

#### 4.5.4. Castor Oil Induced Diarrhea

A previously reported protocol was adopted [45]. Briefly, groups of mice (*n* = 6) were fasted for 18 h, only allowing access to water. The dosing system for each group is as detailed in Section 4.5.3. Diarrhea was triggered by oral administration of 0.5 mL of castor oil to each mouse, 1 h after dosing. Thereafter, each mouse was individually placed in a transparent cage lined with blotting paper. The paper was changed every hour, over four hours of observation time. Occurrence of diarrhea, fecal volume, weight of wet feces, and total fecal content were calculated. Finally, the percentages of fecal output and inhibition of diarrhea (Percent inhibition of defecation) were calculated using the following:

(1) $$\%\;\mathrm{of}\;\mathrm{fecal}\;\mathrm{output}=\frac{\mathrm{Mean}\;\mathrm{faecal}\;\mathrm{weight}\;\mathrm{of}\;\mathrm{each}\;\mathrm{treatment}\;\mathrm{group}}{\mathrm{Mean}\;\mathrm{faecal}\;\mathrm{weight}\;\mathrm{of}\;\mathrm{control}\;\mathrm{group}}\times 100$$

(2) $$\%\;\mathrm{Inhibition}\;\mathrm{of}\;\mathrm{defecation}=\frac{\mathrm{Mo}-M}{\mathrm{Mo}}\times 100$$

where Mo is the mean defecation of control, M is the mean defecation of test sample or standard drug.

#### 4.5.5. Castor Oil Induced Enteropooling

A literature procedure [46] was followed to evaluate intraluminal accumulation. Dosing treatments are as described in Section 4.5.3. One hour following administration of each test dose, 0.5 mL of castor oil was administered orally to each mouse to induce diarrhea. Two hours later, under chloroform anesthesia, the mice were sacrificed, and the small intestine ligated from the pyloric sphincter to ileocecal junction. The small intestinal tract was weighed (g), and the intestinal contents measured by milking into a graduated tube. The intestine was weighed again, and the difference between full and empty intestine determined. The percentage reduction in intestinal secretion and the weight of intestinal contents was estimated using the following formula:

(3) $$\%\;\mathrm{of}\;\mathrm{inhibition}\;\mathrm{using}\;\mathrm{MVSIC}=\frac{\mathrm{MVICC}-\mathrm{MVICT}}{\mathrm{MVICC}}\times 100$$

where MVSIC is the mean volume of the small intestinal content, MVICC is the mean volume of the intestinal content of the control group, and MVICT is the mean volume of the intestinal content of the test group.

#### 4.5.6. Gastrointestinal Motility Test

Gastrointestinal motility testing was conducted according to the method of Carlo et al. [35]. Mice of each group were treated as described in Section 4.5.3. Briefly, to induce diarrhea, 0.5 mL of castor oil was orally administered to each mouse. One hour following administration of each test dose, mice were treated with 1 mL of a charcoal meal (10% charcoal suspension in 5% gum acacia). Afterwards, mice were sacrificed, and the distance traveled by the charcoal meal from the pyloric sphincter to ileocecal junctions was measured (cm). The peristalsis index and percentage of inhibition were determined using the following equation:

(4) $$\mathrm{Peristalsis}\;\mathrm{index}=\frac{\mathrm{Distance}\;\mathrm{travelled}\;\mathrm{by}\;\mathrm{charcoal}\;\mathrm{meal}}{\mathrm{Length}\;\mathrm{of}\;\mathrm{small}\;\mathrm{intestine}}\times 100$$

(5) $$\%\;\mathrm{inhibition}=\frac{\mathrm{Dc}-\mathrm{Dt}}{\mathrm{Dc}}\times 100$$

where Dc is the mean distance travelled in the control group and Dt is the mean distance travelled in the test group.

#### 4.5.7. In Vivo Antidiarrheal Index

The in vivo antidiarrheal index (ADI) was determined using the following equation:

ADI in vivo = 3√ (D freq × G meq × P freq)
(6)

where D freq is the delay in defecation time or diarrhea onset obtained from castor oil diarrheal test by

(7) $$\begin{array}{c} D\;\mathrm{freq}=\\ \frac{\mathrm{mean}\;\mathrm{onset}\;\mathrm{of}\;\mathrm{diarrhoea}\;\mathrm{in}\;\mathrm{the}\;\mathrm{test}\;\mathrm{group}-\mathrm{mean}\;\mathrm{onset}\;\mathrm{of}\;\mathrm{diarrhoea}\;\mathrm{in}\;\mathrm{the}\;\mathrm{control}\times 100}{\mathrm{mean}\;\mathrm{onset}\;\mathrm{of}\;\mathrm{diarrhoea}\;\mathrm{in}\;\mathrm{control}\;\mathrm{group}} \end{array}$$

G meq is the gut meal travel reduction (% of control) obtained from charcoal meal test (% inhibition), and P freq is the purging frequency or reduction in the number of wet stools (as a % of control) obtained from the castor oil diarrheal model (% inhibition of defecation).

### 4.6. In Vitro Cytotoxicity Assay

The cytotoxic activity of *T. leucostaphylum* crude methanol extract and its fractions were investigated using an established naupli Brine shrimp protocol [47]. Brine shrimp eggs were obtained from the Department of Marine Science, University of Chittagong, Bangladesh and were hatched into artificial seawater (sea salt 38 g/L) with constant oxygenation over 48 h under the provision of light to yield naupli. Prior to testing, plant extracts were separately dissolved into marine water with DMSO as cosolvent (<50 μL/5 mL solution) before transfer to a test tube at selected concentrations of 12.5, 25, 50, 100, 200, and 800 µg/L in artificial seawater. The number of viable naupli was analyzed within each test tube after 24 h of incubation at 25–30 °C.

### 4.7. In Silico Studies

#### 4.7.1. Molecular Docking Analysis: Ligand Preparation

The chemical structures of all compounds, detected upon GC-MS analysis of the dichloromethane extract were downloaded from the PubChem compound repository (https://pubchem.ncbi.nlm.nih.gov/). By utilizing the LigPrep (version 42013) tool (Schrodinger suit, LLC New York, NY, USA) embedded in Schrödinger suite-Maestro v11.2, the following parameters were applied: neutralized at pH 7.0 ± 2.0 using Epik 2.2, for minimization OPLS3 force field.

#### 4.7.2. Chemical Compounds Studied in This Article

2,4-Di-*tert*-butylphenol (PubChem CID: 7311); Pentadecene (PubChem CID: 25913); Nonadecene (PubChem CID: 29075); Methyl palmitate (PubChem CID: 8181); Palmitic acid (PubChem CID: 985); Behenic alcohol (PubChem CID: 12620); Methyl lineoleate (PubChem CID: 5284421); Methyl elaidolinolenate (PubChem CID: 5367462); Linoleic acid (PubChem CID: 5280450); and Linolenic acid (PubChem CID: 5280934).

#### 4.7.3. Molecular Docking Analysis: Enzyme/Receptor Preparation

In order to run computational experimental tests, 3D crystallographic structures of various receptors (proteins) were retrieved from the Protein Data Bank; RCSB PDB [48] as follows: M3 muscarinic acetylcholine receptor (PDB ID: 4U14) [49]; 5-HT3 receptor (PDB ID: 5AIN) [50]; Gut inhibitory Phosphodiesterase receptor (PDB ID: 5LAQ) [51,52]; DNA polymerase III subunit alpha (PDB ID: 4JOM); UDP-N-acetylglucosamine-1 carboxyvinyltransferase (PDB ID: 4R7U) [53]. Preparation and refinement of the crystal structures was performed by the Protein Preparation Wizard of Schrödinger suite-Maestro version 11.2. In order to run H-bond optimization and restrain minimization processes, enzymes/proteins must go through a preprocess by assigning charges and bond orders, adding hydrogen to heavy atoms, creating zero order bonds to metals, removing all water molecules followed by generating het states using Epik at pH 7.0 ± 2.0. Restrain minimization was performed using the OPLS3 force field to set a maximum heavy atom RMSD to 0.30 Å.

#### 4.7.4. Molecular Docking Analysis: Grid Standard Precision Docking

Glide was used to carry out the receptor grid generation and molecular docking assay, which was attached to Schrödinger suite-Maestro version 11 [54]. A grid was setup for each protein exerting the consequent default parameters: van der Waals scaling factor 1.00 and charge cut-off value 0.25, subjected to the OPLS3 force field. A cubic box of tangible dimensions centralized on the centroid of the active site residue originated for the respective protein, and the box size was set to 14 Å × 14 Å × 14 Å for docking [55]. The Standard Precision (SP) scoring method of Glide was performed to enact the docking experiments, and for each ligand, only the best scoring pose with docking score was noted.

#### 4.7.5. In Silico ADME Analysis: Determination of Pharmacokinetic Parameters

Pharmacokinetic or Drug-likeness parameters of the identified compounds were estimated according to Lipinski’s rule of five, resolved by the SwissADME online tool (http://www.swissadme.ch/). In accordance with Lipinski’s rule, a compound should behave as a drug if it does not violate more than one of following criteria: (i) molecular weight not more than 500; (ii) H-bond donors ≤ 5; (iii) H-bond acceptors ≤ 10; and (iv) Lipophilicity (LogP) < 5 [56].

## 5. Conclusions

Bioassay of several fractions of *T. leucostaphylum* was undertaken to explore the anti-diarrheal value of this plant and to evaluate its cytotoxic potential. The results suggest that the dichloromethane (DTL) extract has the most potential. During antidiarrheal analysis, all extracts, and particularly the DTL, showed consistent and dose dependent anti-diarrheal efficacy. In addition, computational analysis revealed promising binding affinities of identified constituents (GC-MS) towards different receptors, which proposed antidiarrheal mechanisms for *T. leucostaphylum* at various receptors. The drug like behavior of identified constituents (GC-MS) was tested through ADME/T analysis. Further studies are necessary to isolate and identify the bioactive compounds from *T. leucostaphylum* to more completely characterize its biological activities.

## Figures and Tables

**Table 1:** List of chemical compounds in the dichloromethane (DTL) extract of *T. leucostaphylum*, identified by GC-MS.

Sl. No.	Retention Time (min)	Peak Area (%)	Name of Compound	Molecular Formula
1	24.75	2.79	2,4-Di-*tert*-butylphenol	C_14_H_22_O
2	26.85	2.21	Pentadecene	C_15_H_30_
3	31.30	3.20	Nonadecene	C_19_H_38_
4	34.04	16.41	Methyl palmitate	C_17_H_34_O_2_
5	34.85	16.62	Palmitic acid	C_16_H_32_O_2_
6	35.80	2.66	Behenic alcohol	C_22_H_46_O
7	38.84	12.14	Methyl lineoleate	C_19_H_34_O_2_
8	39.04	20.75	Methyl elaidolinolenate	C_19_H_32_O_2_
9	40.28	10.60	Linoleic acid	C_18_H_32_O_2_
10	40.51	12.63	Linolenic acid	C_18_H_30_O_2_

**Table 2:** Effect of methanol extract of *T. leucostaphylum* leaves and its derived fractions on castor oil induced diarrhea in mice.

Dose Treatment (mg/kg, p.o)	Onset Diarrhea (min)	Total No of Dry Faces	Total No of Wet Faces	Weight (mg) of Dry Faces	Weight (mg) of Wet Faces	% Inhibition of Defecation
Control	14.17 ± 1.12	9.92 ± 0.34	6.92 ± 0.33	0.48 ± 0.002	0.37 ± 1.23	−
Loperamide 3	93.39 ± 2.38 ***	3.6 ± 0.23 ***	1.7 ± 0.24 ***	0.13 ± 0.08 **	0.09 ± 0.07 *	75.44%
NTL 100	54.50 ± 1.34 ***	9.67 ± 0.76	3.63 ± 0.22 ***	0.88 ± 0.02	0.53 ± 0.03	47.54%
NTL 200	66.16 ± 1.82 ***	5.0 ± 1.63 ***	3.0 ± 0.16 ***	0.64 ± 0.16	0.36 ± 0.11 *	56.64%
NTL 400	84.67 ± 2.27 ***	4.0 ± 1.00 ***	2.0 ± 0.18 ***	0.72 ± 0.18 **	0.26 ± 0.08 ***	71.09%
DTL 100	75.33 ± 0.56 ***	2.33 ± 0.80 ***	2.21 ± 0.02 ***	0.33 ± 0.04 ***	0.30 ± 0.02 ***	68.06%
DTL 200	85.67 ± 1.66 ***	1.6 ± 1.06 ***	1.5 ± 0.05 ***	0.21 ± 0.05 **	0.28 ± 0.11 *	76.15%
DTL 400	99.5 ± 0.12 ***	1.5 ± 0.95 ***	0.83 ± 0.13 ***	0.32 ± 0.13 **	0.21 ± 0.15 ***	88.01%
BTL 100	50.5 ± 0.85 ***	7.17 ± 0.50 *	4.10 ± 0.13	0.78 ± 0.02 *	0.51 ± 0.01	40.75%
BTL 200	66 ± 1.81 ***	5.3 ± 0.73 ***	3.50 ± 0.09 **	0.61 ± 0.09	0.37 ± 0.06 *	49.42%
BTL 400	77.6 ± 1.39 ***	4.17 ± 0.43 ***	3.33 ± 0.02 ***	0.51 ± 0.02 **	0.28 ± 0.15 *	51.87%
MTL 100	47.00 ± 1.90 ***	10.17 ± 0.48	4.23 ± 1.16 *	1.25 ± 0.06 **	1.00 ± 0.05 *	38.87%
MTL 200	54.34 ± 1.30 ***	6.67 ± 1.60 **	3.83 ± 0.06 **	0.38 ± 0.06 *	0.81 ± 0.05	44.65%
MTL 400	64.37 ± 2.40 ***	6.16 ± 1.11 **	3.67 ± 0.06 ***	0.60 ± 0.05	0.47 ± 0.12 **	46.96%

Results are presented as Mean ± SEM (*n* = 6); One Way Analysis of Variance (ANOVA) followed by Dunnett’s Multiple Comparison Test; * *p* < 0.05, ** *p* < 0.01, *** *p* < 0.001 were considered significant compared to the control sample. NTL = *n*-hexane fraction of *T. leucostaphylum*, DTL = dichloromethane fraction of *T. leucostaphylum*, BTL = *n*-butanol fraction of *T. leucostaphylum*, MTL = methanol extract of *T. leucostaphylum*.

**Table 3:** Effect of methanol extract of *T. leucostaphylum* leaves and its derived fractions on castor oil induced intraluminal fluid accumulation in mice.

Treatment (mg/kg, p.o)	MWSIC (g)	% Inhibition (Using MWSIC)	MVSIC (mL)	% Inhibition (Using MVSIC)
Control	0.72 ± 0.02	−	0.68 ± 0.02	−
Loperamide 3	0.18 ± 0.01 ***	75%	0.16 ± 0.01 ***	76.5%
NTL 100	0.48 ± 0.01 **	33.3%	0.48 ± 0.02 *	29.41%
NTL 200	0.40 ± 0.03 **	43.3%	0.43 ± 0.04 **	36.3%
NTL 400	0.26 ± 0.02 ***	63.3%	0.26 ± 0.14 ***	61.02%
DTL 100	0.22 ± 0.01 ***	69.4%	0.36 ± 0.02 **	47.05%
DTL 200	0.18 ± 0.01 ***	74.86%	0.18 ± 0.06 ***	73.38%
DTL 400	0.04 ± 0.03 ***	93.75%	0.06 ± 0.05 ***	91.17%
BTL 100	0.56 ± 0.02 **	22.22%	0.50 ± 0.01 *	26.47%
BTL 200	0.50 ± 0.02 **	30.6%	0.47 ± 0.03 *	30.7%
BTL 400	0.36 ± 0.01 ***	49.8%	0.36 ± 0.01 **	46.03%
MTL 100	0.70 ± 0.01	2.78%	0.65 ± 0.024	4.41%
MTL 200	0.67 ±0.020	5.83%	0.645 ± 0.04	5.14%
MTL 400	0.55 ± 0.018	22.5%	0.530 ± 0.14	22.05%

Values are expressed as mean ± SEM (*n* = 6); One-way analysis of variance (ANOVA) suggested by Dunnett’s test. * *p* < 0.05, ** *p* < 0.01, *** *p* < 0.001 were considered significant compared to the control. NTL = *n*-hexane fraction of *T. leucostaphylum*, DTL = dichloromethane fraction of *T. leucostaphylum*, BTL = *n*-butanol fraction of *T. leucostaphylum*, MTL = methanol extract of *T. leucostaphylum*. MWSIC = Mean Weight of the Small Intestinal Content, MVSIC = Mean Volume of the Small Intestinal Content.

**Table 4:** Effect of methanol extract of *T. leucostaphylum* leaves and its derived fractions on castor oil induced intestinal transit in mice (using a charcoal meal as a marker).

Treatment (mg/kg, p.o)	Mean Distance Travelled by Charcoal (cm)	Mean Length of Ligated S.I. (cm)	Peristalsis Index	% of Inhibition
Control	46.72 ± 0.89	51.60 ± 0.92	90.54%	−
Loperamide 3	11.59 ± 0.45 ***	51.83 ± 1.32 *	22.36%	75.19%
NTL 100	18.07 ± 0.46 ***	49.67 ± 0.88	36.68%	61.28%
NTL 200	16.65 ± 2.89 ***	50.33 ± 1.28 ***	33.08%	64.36%
NTL 400	7.467 ± 2.31 ***	49.5 ± 2.68	15.08%	78.31%
DTL 100	15.67 ± 0.88 ***	49.67 ± 0.67 *	31.55%	66.46%
DTL 200	13.00 ± 2.46 ***	50.167 ± 1.30 ***	25.91%	72.17%
DTL 400	7.34 ± 2.760 ***	49.67 ± 1.36 ***	14.77%	84.28%
BTL 100	18.52 ± 1.18 ***	50.83 ± 0.79	36.44%	60.34%
BTL 200	17.18 ± 1.19 ***	52.5 ± 1.09 *	32.72%	62.22%
BTL 400	14.27 ± 0.70 ***	51.83 ± 1.08 **	27.53%	69.45%
MTL 100	35.17 ± 1.22	50.67 ± 0.67 **	69.41%	24.72%
MTL 200	32.66 ± 2.17 *	52.67 ± 1.05	62.08%	30.09%
MTL 400	29.34 ± 2.51 **	51.67 ± 1.31 **	56.77%	37.2%

Data are presented as mean ± SEM (*n* = 6); One Way Analysis of Variance (ANOVA) followed by Dunnett’s Multiple Comparison Test; * *p* < 0.05, ** *p* < 0.01, *** *p* < 0.001 were considered significant compared to the control. NTL = *n*-hexane fraction of *T. leucostaphylum*, DTL = dichloromethane fraction of *T. leucostaphylum*, BTL = *n*-butanol fraction of *T. leucostaphylum*, MTL = methanol extract of *T. leucostaphylum*.

**Table 5:** In vivo antidiarrheal index value of the methanol extract of *T. leucostaphylum* leaves and its derived fractions.

Treatment (mg/kg, p.o)	Delay in Defecation Time (Dfreq)	Gut Meal Travel Reduction (Gmeq)	Purging Frequency (Pfreq)	Antidiarrheal Index ADI
Control	−	−	−	−
Loperamide 3	559.07	75.19	75.44	146.92
NTL 100	143.47	61.28	47.54	93.95
NTL 200	366.95	64.36	56.64	110.18
NTL 400	497.53	78.31	71.09	140.44
DTL 100	361.05	66.46	68.06	124.98
DTL 200	504.59	72.17	76.15	140.49
DTL 400	602.19	84.28	88.01	164.69
BTL 100	178.76	60.34	40.75	85.75
BTL 200	365.77	62.22	49.42	103.99
BTL 400	447.64	69.45	51.87	117.27
MTL 100	231.69	24.72	38.87	60.61
MTL 200	283.49	30.09	44.65	72.49
MTL 400	354.27	37.2	46.96	85.22

NTL = *n*-hexane fraction of *T. leucostaphylum*, DTL = dichloromethane fraction of *T. leucostaphylum*, BTL = *n*-butanol fraction of *T. leucostaphylum*, MTL = methanol extract of *T. leucostaphylum*.

**Table 6:** Docking scores of the bioactive compounds identified in the dichloromethane extract (DTL) of *T. leucostaphylum*.

Compound Name	Docking Score				
	4U14	5AIN	5LAQ	4JOM	4R7U
2,4-Di-*tert*-butylphenol	−8.14	−5.89	−8.37	−4.92	−4.85
Pentadecene	−0.08	+2.61	+1.45	+3.81	+3.46
Nonadecene	−0.84	+0.96	−0.75	+3.53	+2.18
Methyl palmitate	−2.16	−0.27	−1.69	+1.79	+1.80
Palmitic acid	−1.54	+0.09	−1.63	+1.28	+1.84
Behenic alcohol	−5.23	−3.28	−5.66	−1.15	−2.78
Methyl lineoleate	−3.75	−1.75	−3.09	+0.68	+0.67
Methyl elaidolinolenate	−3.96	−2.03	−2.39	+1.29	−0.44
Linoleic acid	−3.85	−0.72	−3.47	+0.73	+0.25
Linolenic acid	−2.94	−1.00	−3.99	−0.05	+0.01
Reference drug	−7.28	−	−5.93	−4.17	−4.57

Docking scores in Kcal/mol; bold text indicates the highest score.

**Table 7:** Binding interactions of (2,4-di-*tert*-butylphenol) as identified in the molecular docking study.

Compound	Antidiarrheal Receptors (PDB)	Hydrogen Bond Interactions		Hydrophobic Interactions	
		Amino Acid Residue	Distance (Å)	Amino Acid Residue	Distance (Å)
**2,4-Di-*tert*-butylphenol**	4U14	ASN-507	2.39	ALA-238	4.23
				TRP-503	4.52
	5AIN	−	−	TYR-186	4.23
				TRP-145	4.53
				VAL-146	4.96
				ILE-116	4.21
				CYS-189	4.96
				CYS-188	5.43
					4.96
				MET-114	5.02
	5LAQ	GLN-615	1.86	ILE-582	4.57
					2.72
				PHE-586	5.00
				PHE-618	4.20
					4.02
	4JOM	LYS-543	2.40	−	
		PHE-556	1.77		
	4R7U	LYS-23	2.27	VAL-164	4.98
		ASN-24	2.41	ARG-121	3.79
				ALA-93	4.67

**Table 8:** Physicochemical properties of the compounds identified in the dichloromethane extract (DTL) of *T. leucostaphylum*.

Compound	MW	HBA	HBD	Log P	Lipinski’s Violations
Rule	<500	<10	≤5	≤5	≤1
2,4-Di-*tert*-butylphenol	206.32	1	1	3.99	0
Pentadecene	210.40	0	0	6.08	1
Nonadecene	266.51	0	0	7.56	1
Methyl palmitate	270.45	2	0	5.54	1
Palmitic acid	256.42	2	1	5.20	1
Behenic alcohol	326.60	1	1	7.61	1
Methyl lineoleate	294.47	2	0	5.69	1
Methyl elaidolinolenate	292.46	2	0	5.55	1
Linoleic acid	280.45	2	1	5.45	1
Linolenic acid	278.43	2	1	5.09	1

MW, Molecular weight (g/mol); HBA, Hydrogen bond acceptor; HBD, Hydrogen bond donor; Log P, Lipophilicity.

## Supplementary Materials

The following are available online. Figure S1: 3D Protein-Ligand complex for (A) Pentadecene, (B) Nonadecene, (C) Methyl palmitate, (D) Palmitic acid, (E) Behenic alcohol, (F) Methyl lineoleate, (G) Methyl elaidolinolenate, (H) Acidelinoleique, (I) Linolenic acid docked to the M3 muscarinic acetylcholine receptor (PDB ID: 4U14); Figure S2: 3D Protein-Ligand complex for (A) Pentadecene, (B) Nonadecene, (C) Methyl palmitate, (D) Palmitic acid, (E) Behenic alcohol, (F) Methyl lineoleate, (G) Methyl elaidolinolenate, (H) Acidelinoleique, (I) Linolenic acid docked to the 5-HT3 receptor (PDB ID: 5AIN); Figure S3: 3D Protein-Ligand complex for (A) Pentadecene, (B) Nonadecene, (C) Methyl palmitate, (D) Palmitic acid, (E) Behenic alcohol, (F) Methyl lineoleate, (G) Methyl elaidolinolenate, (H) Acidelinoleique, (I) Linolenic acid docked to the gut inhibitory Phosphodiesterase receptor (PDB ID: 5LAQ); Figure S4: 3D Protein-Ligand complex for (A) Pentadecene, (B) Nonadecene, (C) Methyl palmitate, (D) Palmitic acid, (E) Behenic alcohol, (F) Methyl lineoleate, (G) Methyl elaidolinolenate, (H) Acidelinoleique, (I) Linolenic acid docked the DNA polymerase III subunit alpha (PDB ID: 4JOM); Figure S5: 3D Protein-Ligand complex for (A) Pentadecene, (B) Nonadecene, (C) Methyl palmitate, (D) Palmitic acid, (E) Behenic alcohol, (F) Methyl lineoleate, (G) Methyl elaidolinolenate, (H) Acidelinoleique, (I) Linolenic acid docked the UDP-N-acetylglucosamine-1-carboxyvinyltransferase (PDB ID: 4R7U).

## Abbreviations

ADME/T: Absorption, distribution, metabolism, excretion/Toxicity; ADI: antidiarrheal index; BTL: butanol extract of Tetrastigma leucostaphylum; DMSO: dimethyl sulfoxide; DTL: dichloromethane extract of Tetrastigma leucostaphylum; GC-MS: Gas chromatography and Mass Spectroscopy; MTL: methanol extract of Tetrastigma leucostaphylum; MVSIC: mean volume of the small intestinal content; MVICC: mean volume of the intestinal content of the control group; MVICT: mean volume of the intestinal content of the test group; NTL: *n*-hexane extract of Tetrastigma leucostaphylum; TIC: Total ionic chromatogram; TL: Tetrastigma leucostaphylum; VCF: Village Common Forest; WHO; World Health Organization; 5-HT3: 5-hydroxy tryptophan.
