# Supplementary material for: Evaluation of Various Solvent Extracts of Tetrastigma leucostaphylum (Dennst.) Alston Leaves, a Bangladeshi Traditional Medicine Used for the Treatment of Diarrhea
Source: Molecules. 2020 Oct 28;25(21):4994. doi: 10.3390/molecules25214994 (PMC7662597; doi:10.3390/molecules25214994)
Supplement: Supplementary file 1 [file molecules-25-04994-s001.pdf]

Research Article

Evaluation of Various Solvent Extracts of *Tetrastigma leucostaphylum* (Dennst.) Alston ex Mabb leaves, A Bangladeshi Traditional Medicine used for the Treatment of Diarrhea

Sajib Rudra[1, ¶], Md. Adnan[2, ¶], Nazim Uddin Emon[3], Afroza Tahamina[4], Mohammad Shakil[1], Md. Helal Uddin Chowdhury[1], Mohammad Omar Faruque[1], James W. Barlow[5], Sheikh Bokhtear Uddin[1,*]

1 Ethnobotany and Pharmacognosy Lab, Department of Botany, University of Chittagong, Chittagong-4331, Bangladesh
2 Department of Bio-Health Technology, Kangwon National University, Chuncheon 24341, Republic of Korea;
3 Department of Pharmacy, International Islamic University Chittagong, Chittagong 4318, Bangladesh,
4 Beijing Advanced Innovation Center for Food Nutrition and Human Health, Beijing Technology and Business University 100048;
5 Department of Chemistry, Royal College Surgeons, Dublin-D02YN77, Ireland.

¶ These authors contributed equally to this work

* Correspondence: Sheikh Bokhtear Uddin; Email: bokhtear@cu.ac.bd; Tel: +8801711065377

Running title: Antidiarrheal activities of *Tetrastigma leucostaphylum* (Dennst.)

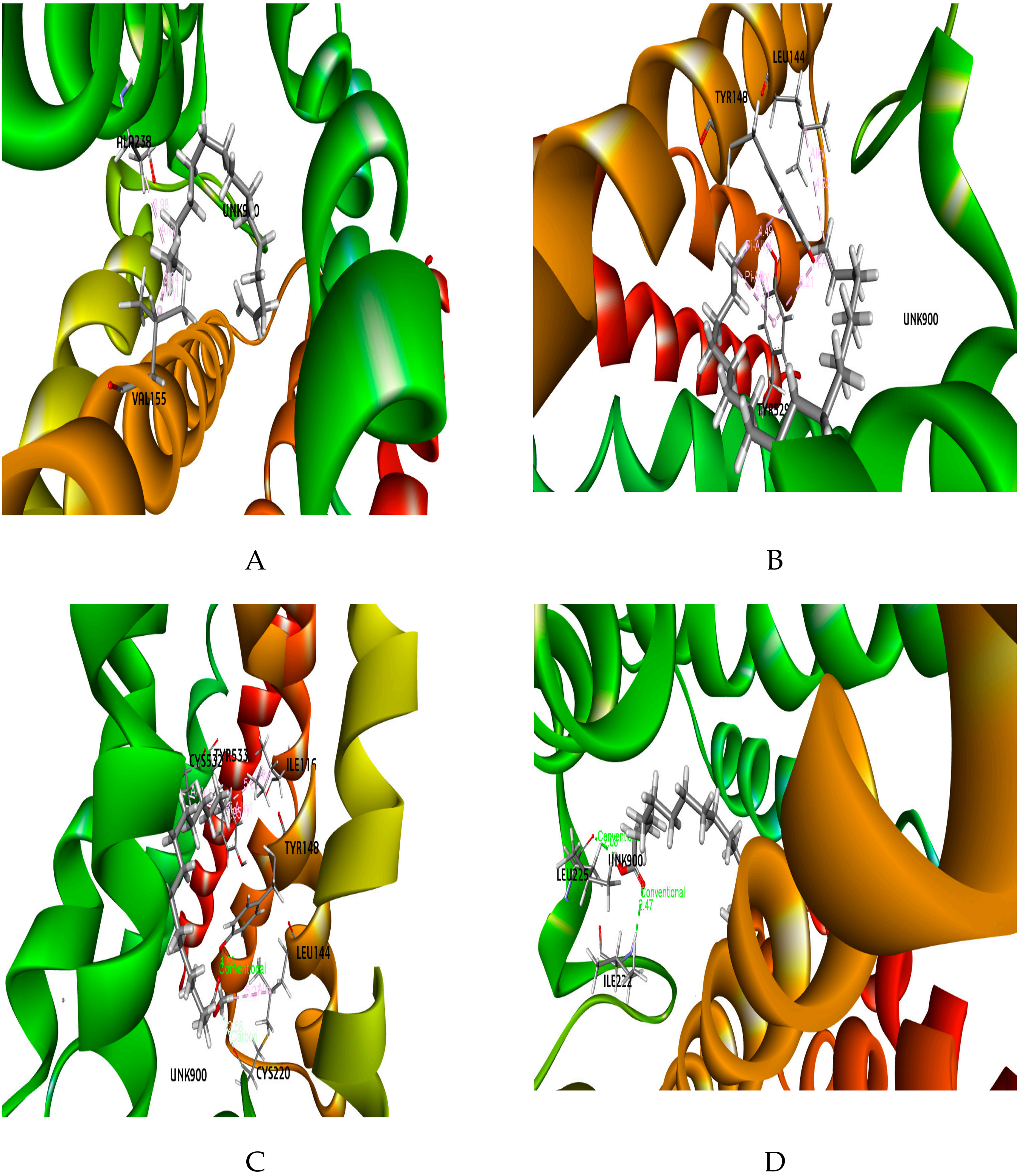
VAL155
LEU144
TYR148
UNK900
TYR533
ILE116
TYR148
LEU144
Conventional
UNK900
CYS220
LEU225
UNK900
Conventional
2.47
ILE222
A
B
C
D

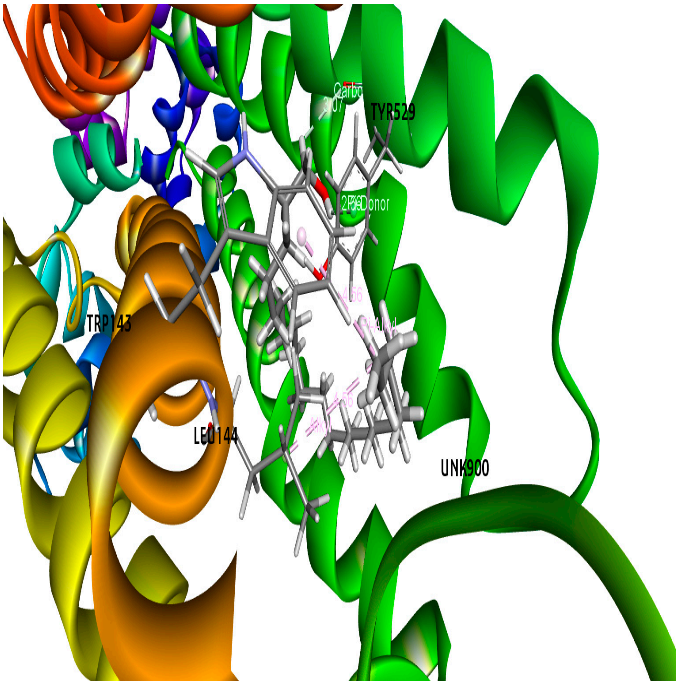
TYR529
TRP143
LEU144
UNK900

E

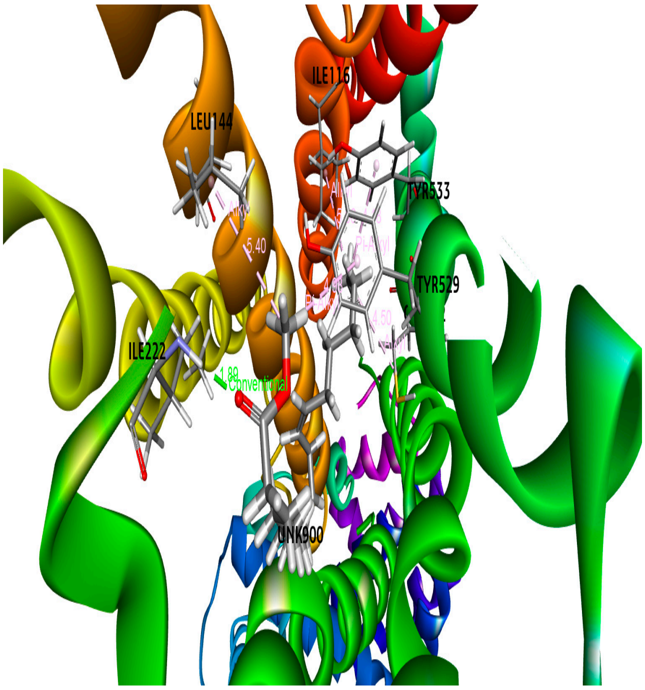
ILE116
LEU144
TYR533
TYR529
ILE222
UNK900

F

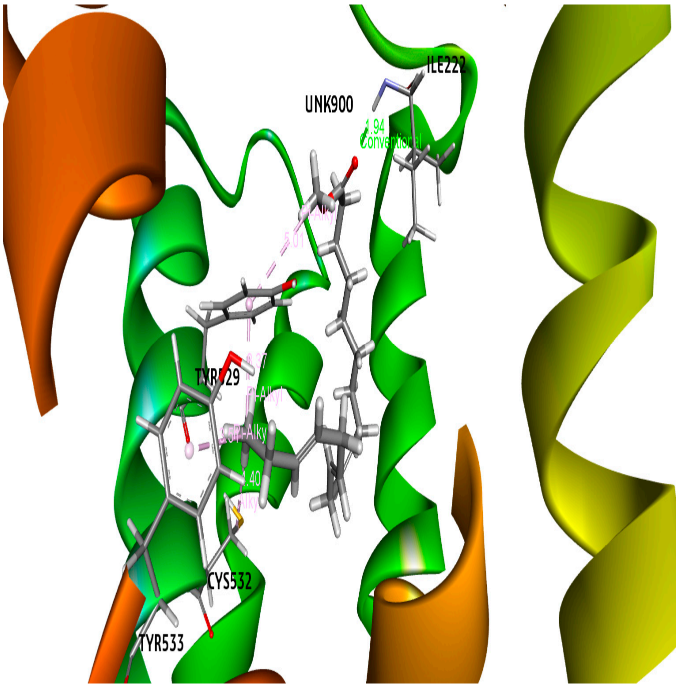
ILE222
UNK900
CYS532
TYR533

G

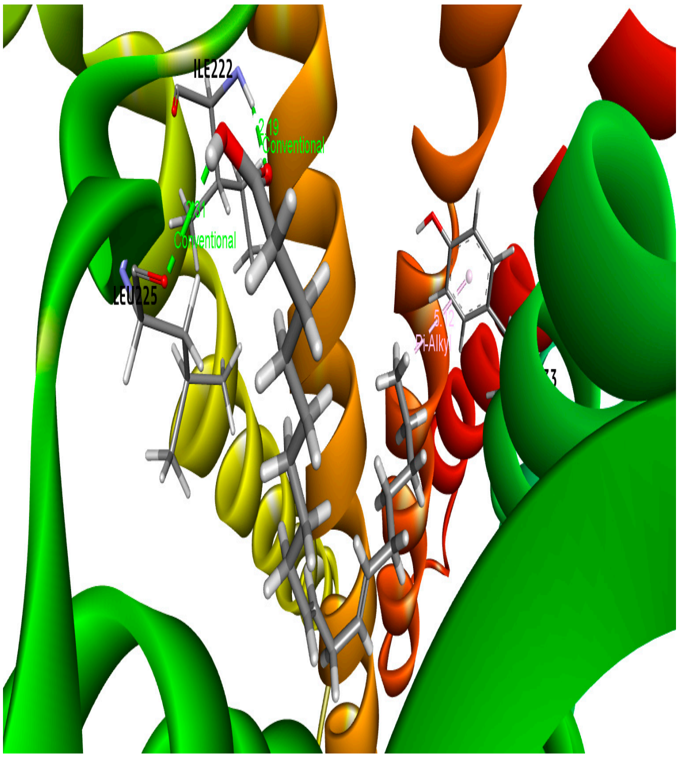
ILE222
LEU225

H

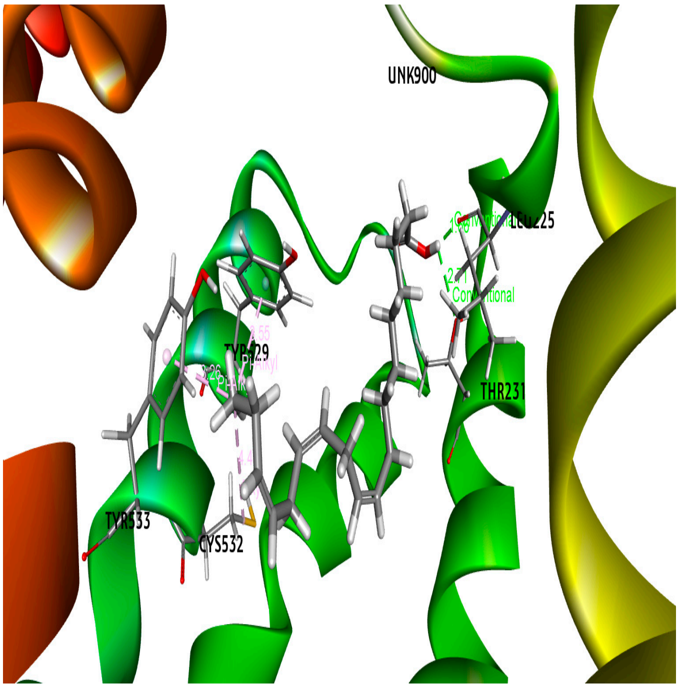

I

Figure 1. 3D Protein-Ligand complex for (A) Pentadecene, (B) Nonadecene, (C) Methyl palmitate, (D) Palmitic acid, (E) Behenic alcohol, (F) Methyl lineoleate, (G) Methyl elaidolinolenate, (H) Acidelinoleique, (I) Linolenic acid docked to the M3 muscarinic acetylcholine receptor (PDB ID : 4U14).

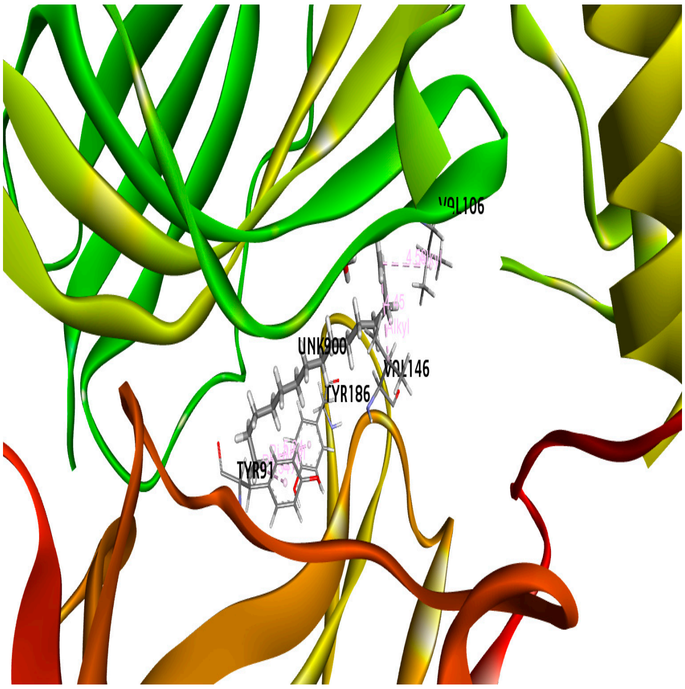
VAL106
UNK900
VAL146
TYR186
TYR91
Alkyl

A

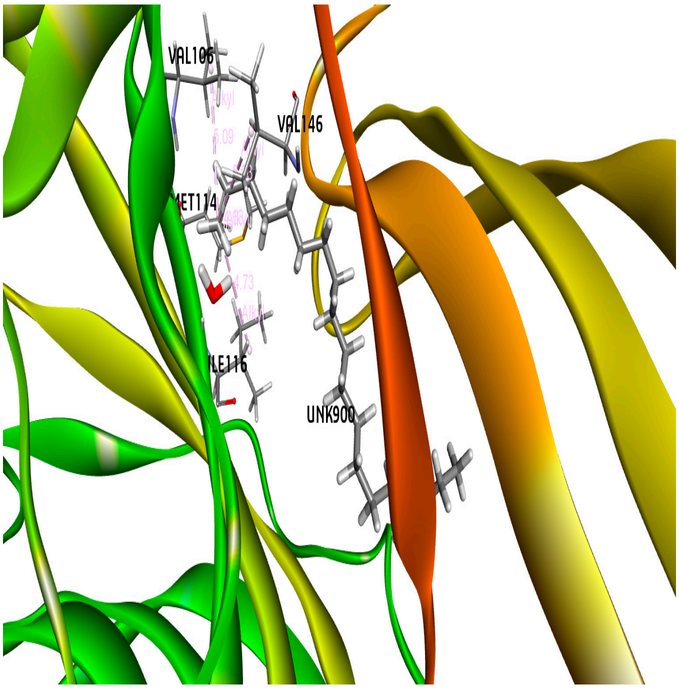
VAL106
VAL146
UNK900

B

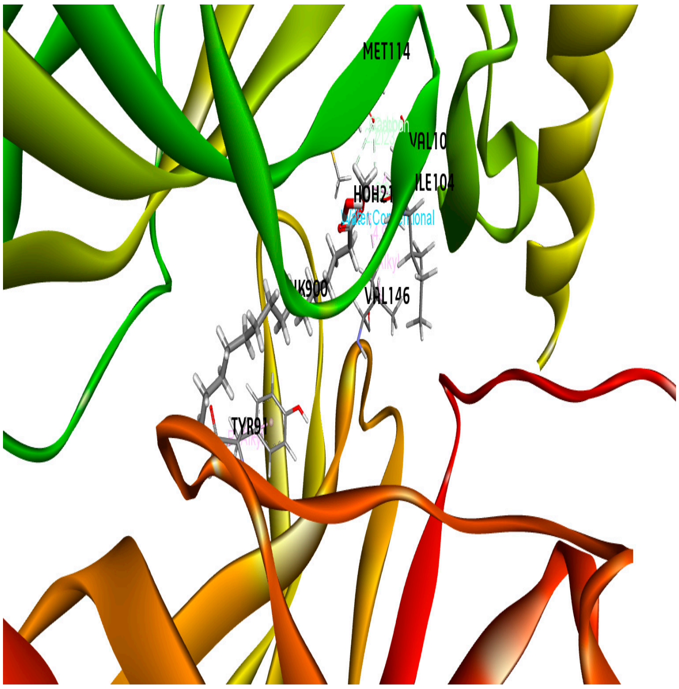
MET114
VAL146
TYR91

C

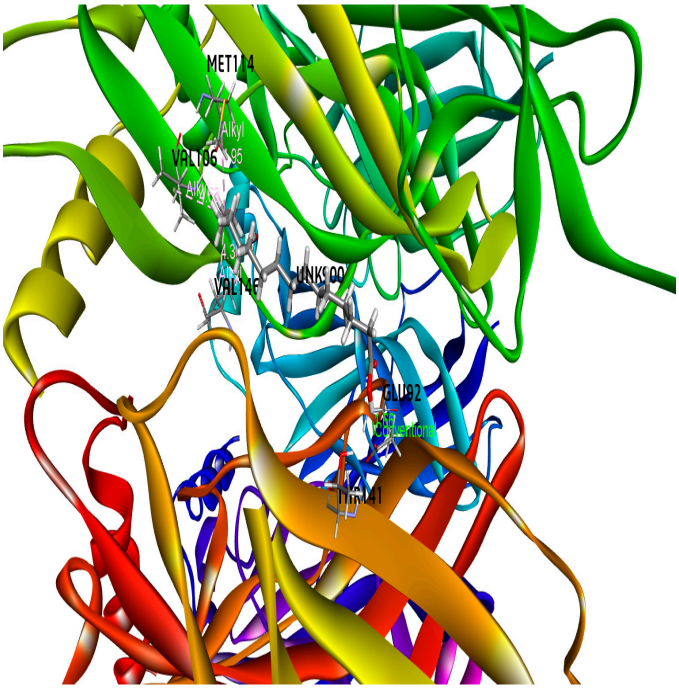
MET114
VAL106
Alkyl
GLU92

D

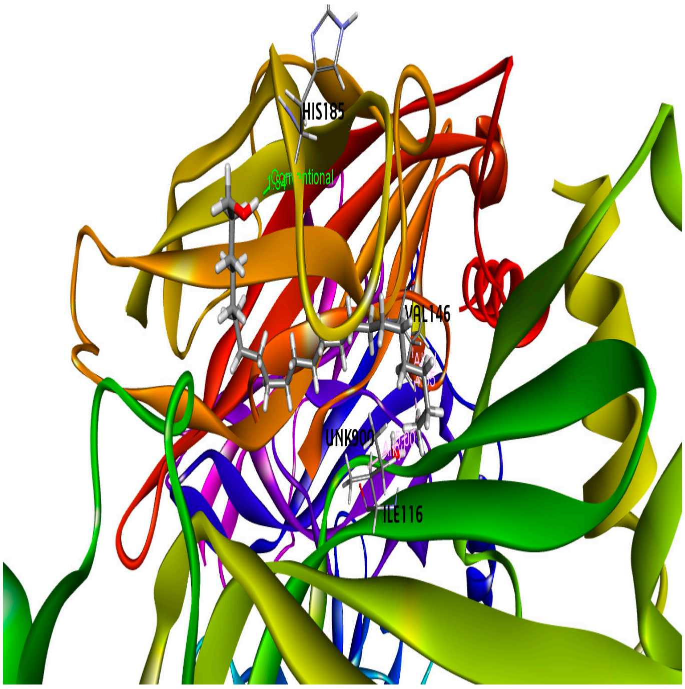
HIS185
VAL146
ILE116

E

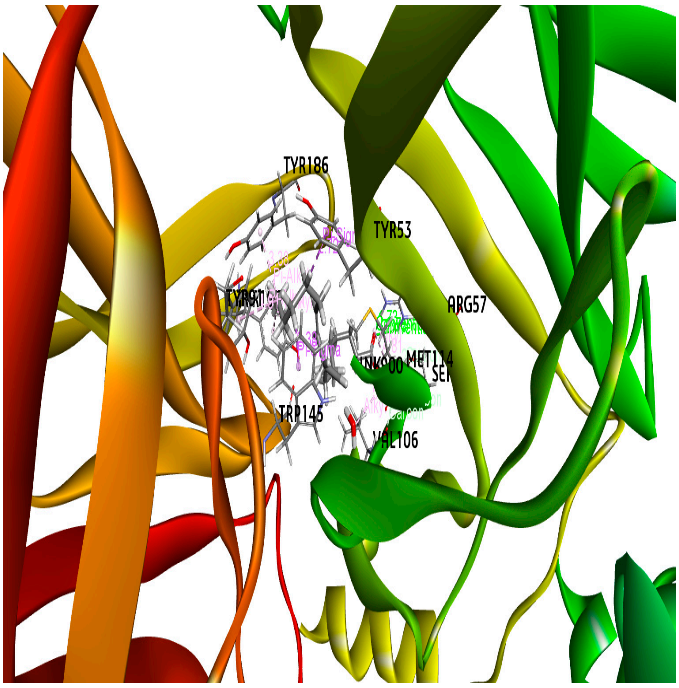
TYR186
TYR53
ARG57
MET114
TRP145
VAL106

F

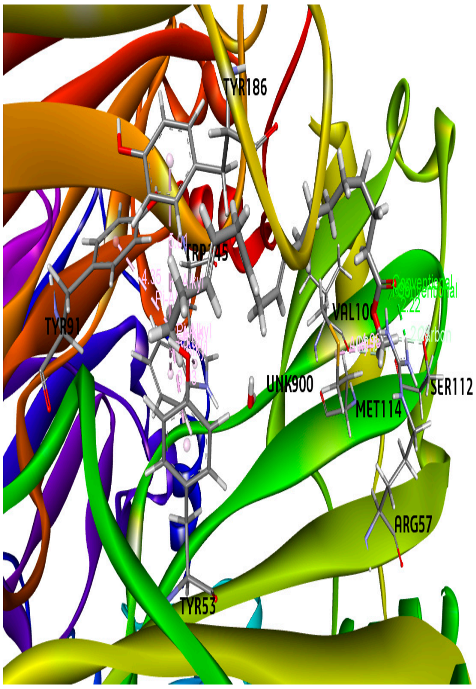
TYR186
TRP145
TYR91
UNK900
MET114
SER112
ARG57
TYR53

G

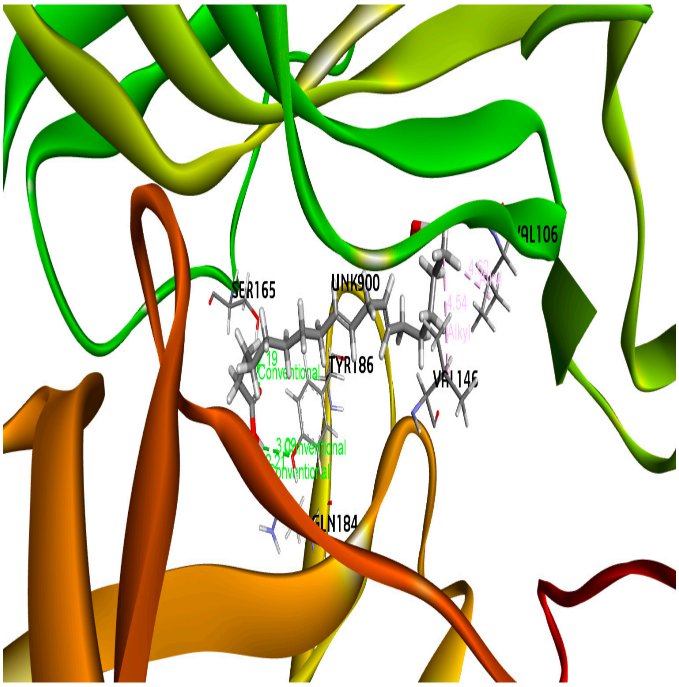
VAL106
SER165
UNK900
TYR186
GLN184

H

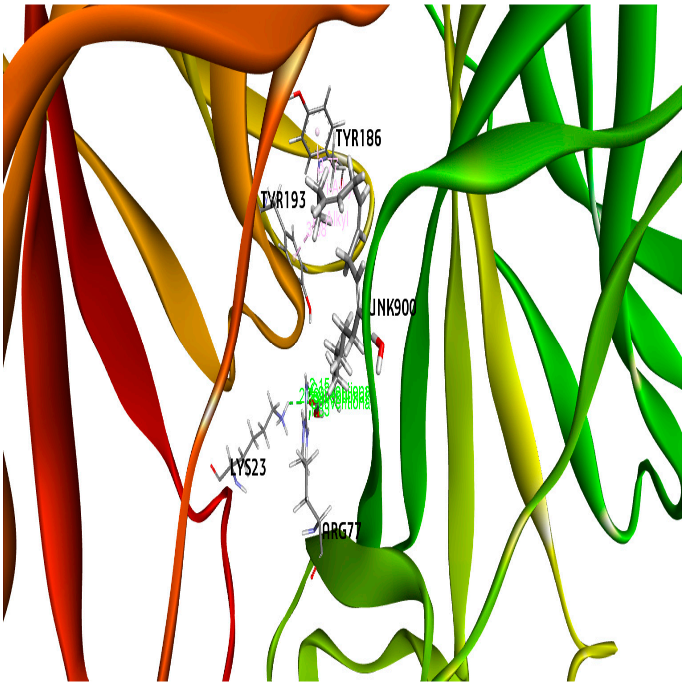

I

Figure 2. 3D Protein-Ligand complex for (A) Pentadecene, (B) Nonadecene, (C) Methyl palmitate, (D) Palmitic acid, (E) Behenic alcohol, (F) Methyl lineoleate, (G) Methyl elaidolinolenate, (H) Acidelinoleique, (I) Linolenic acid docked to the 5-HT3 receptor (PDB ID : 5AIN).

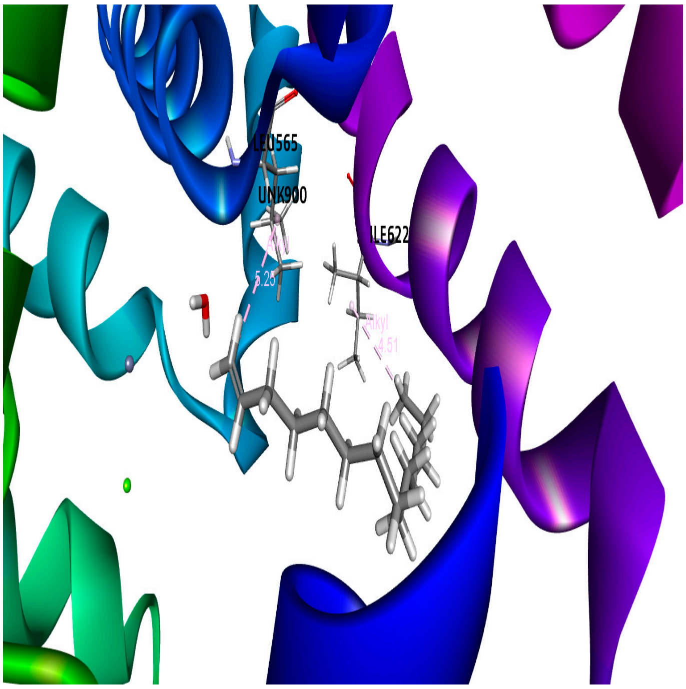
LEU565
UNK900
ILE622

A

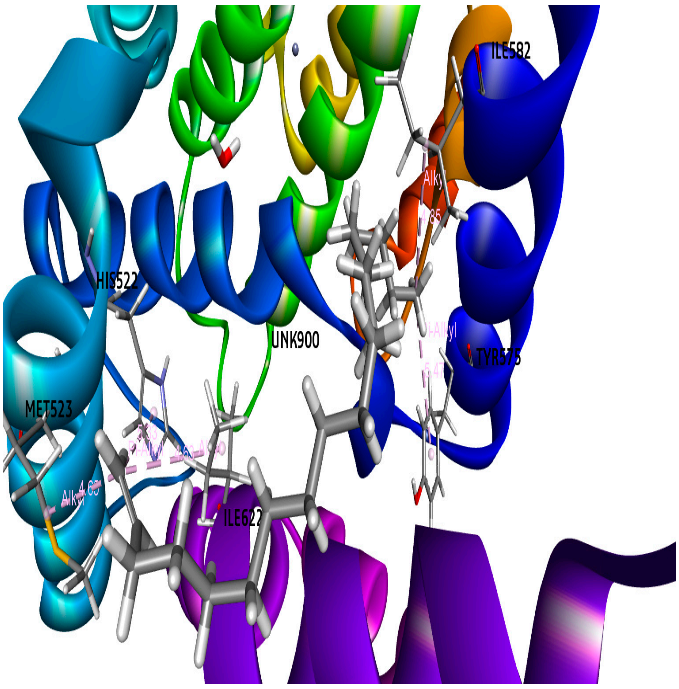
ILE582
HIS522
UNK900
MET523
ILE622

B

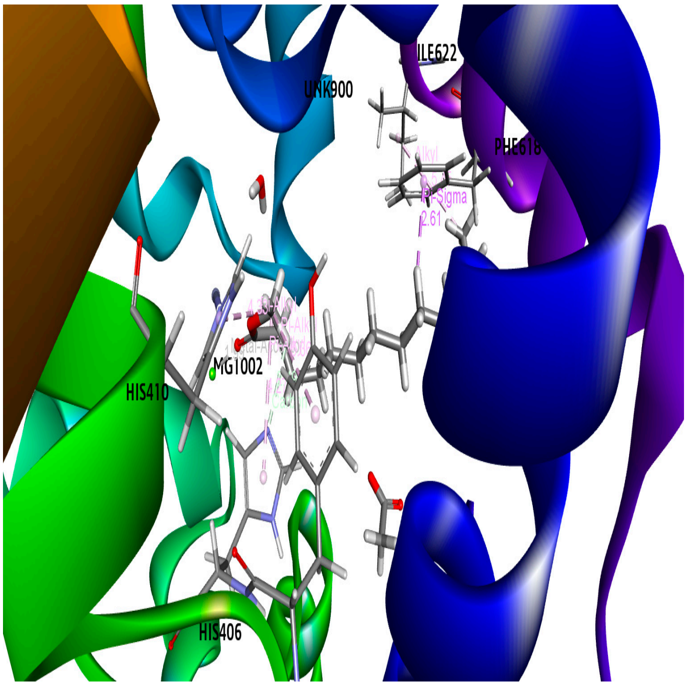
ILE622
UNK900
PHE618
MG1002
HIS410
HIS406

C

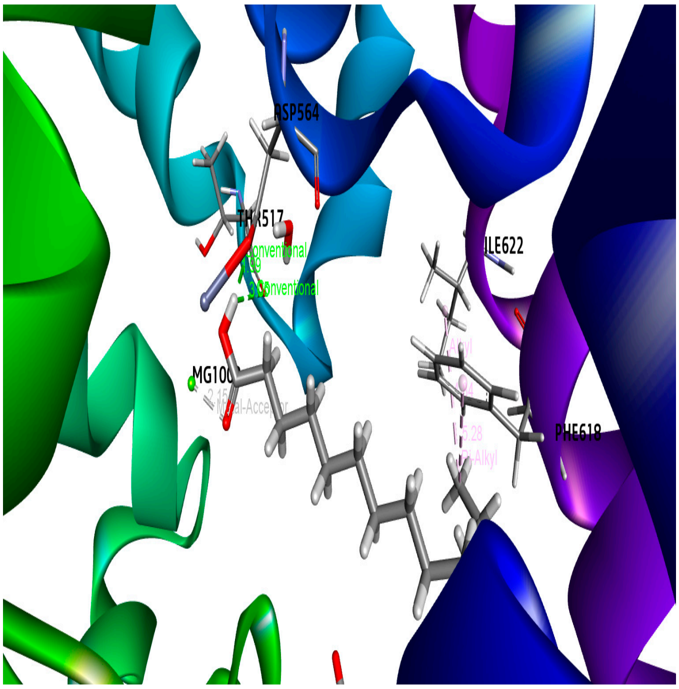
ILE622
PHE618

D

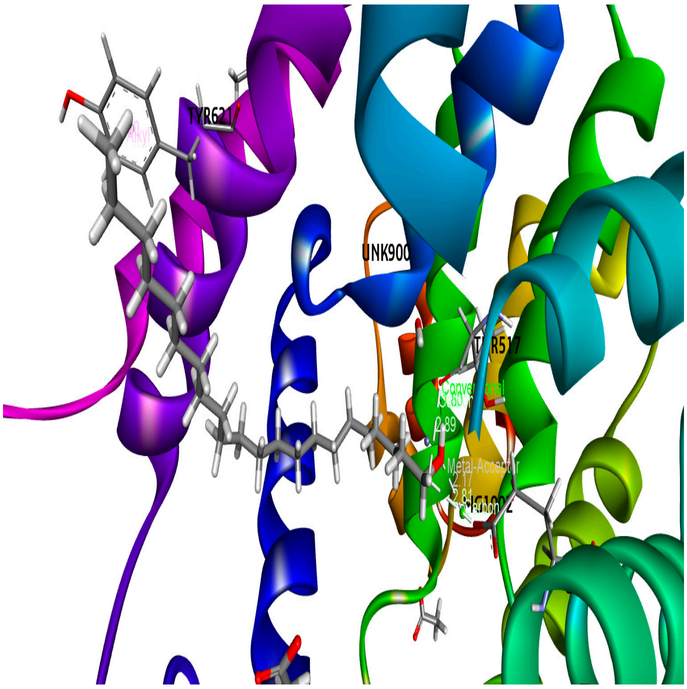
TYR621
UNK900
THR517
MG1002

E

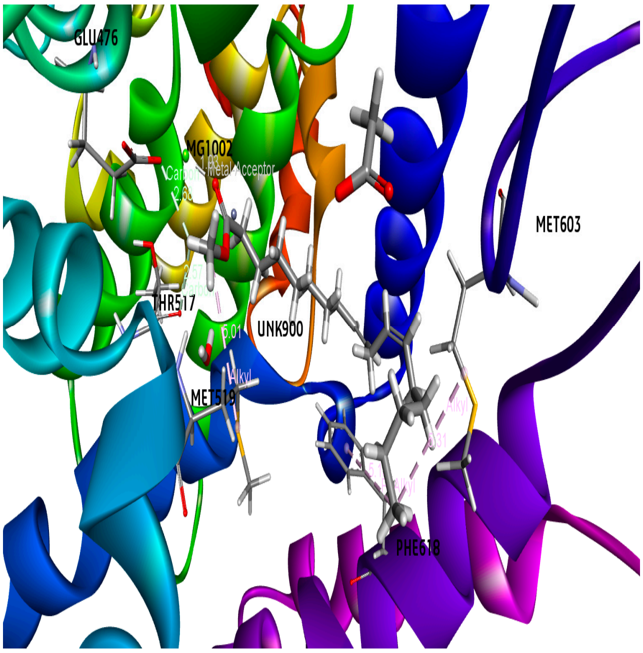
GLU476
MG1002
MET603
THR517
UNK900
MET519
PHE618

F

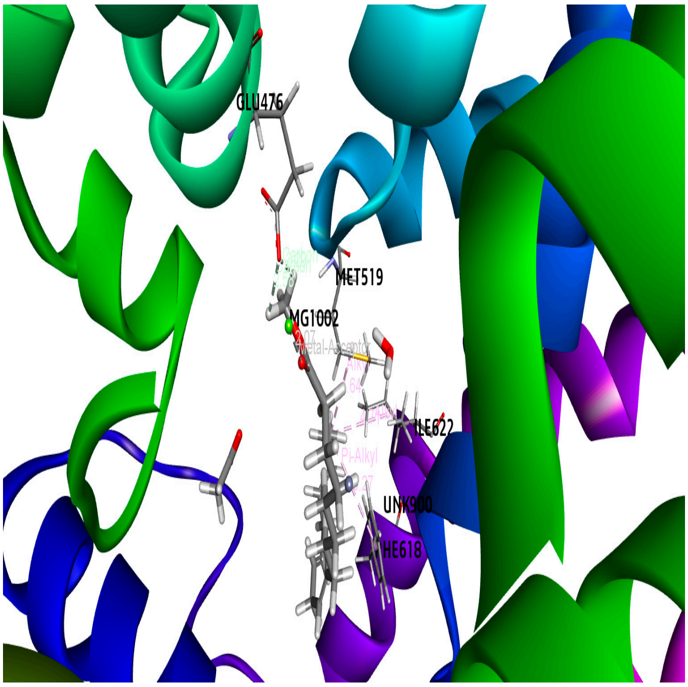
GLU476
MET519
MG1002
ILE622
UNK900

G

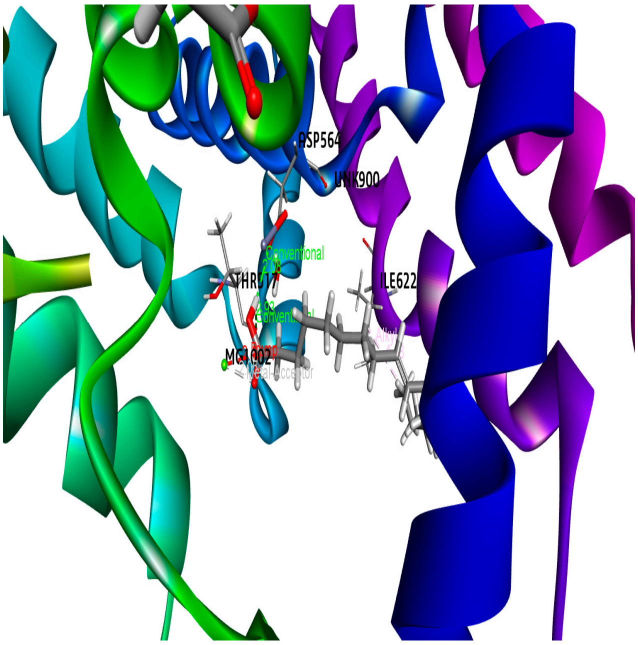
UNK900
ILE622

H

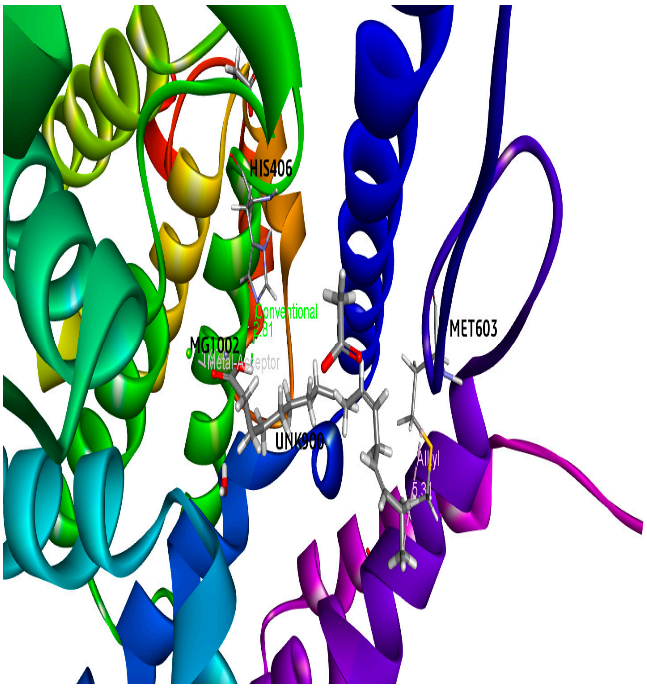

I

Figure 3. 3D Protein-Ligand complex for (A) Pentadecene, (B) Nonadecene, (C) Methyl palmitate, (D) Palmitic acid, (E) Behenic alcohol, (F) Methyl lineoleate, (G) Methyl elaidolinolenate, (H) Acidelinoleique, (I) Linolenic acid docked to the gut inhibitory Phosphodiesterase receptor (PDB ID : 5LAQ).

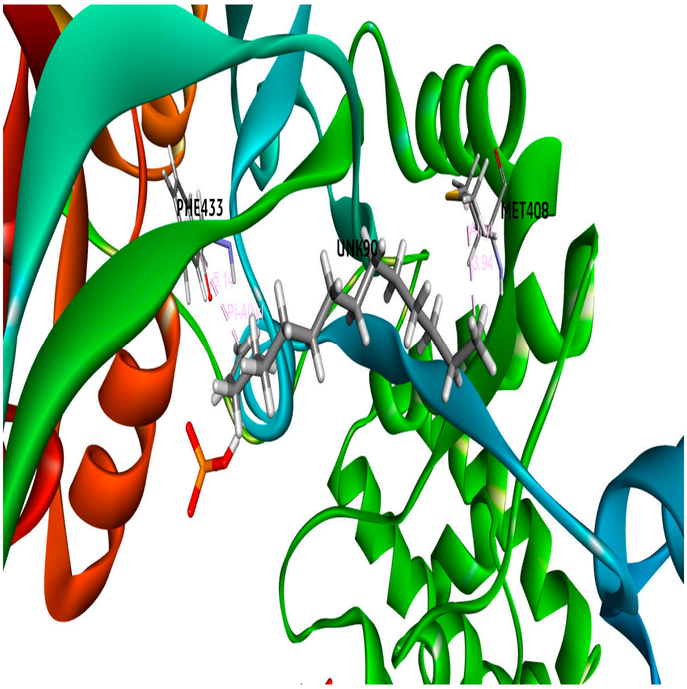
PHE433
UNK90
MET408

A

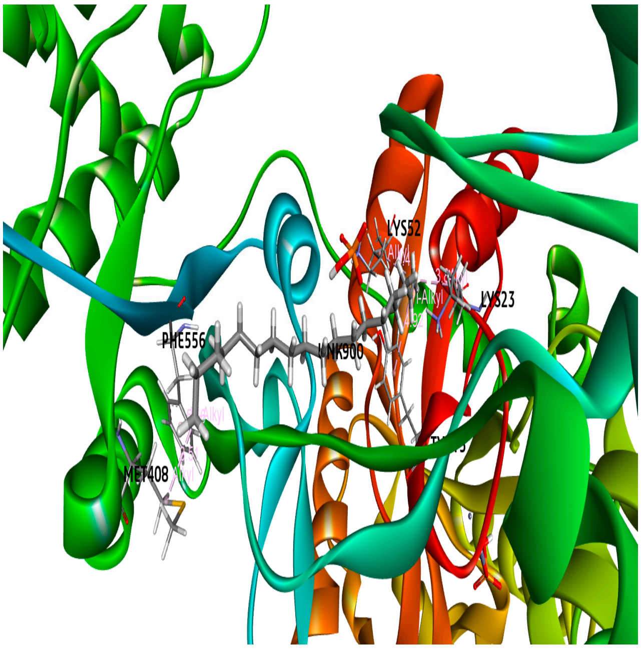
LYS52
LYS23
PHE556
UNK900
MET408

B

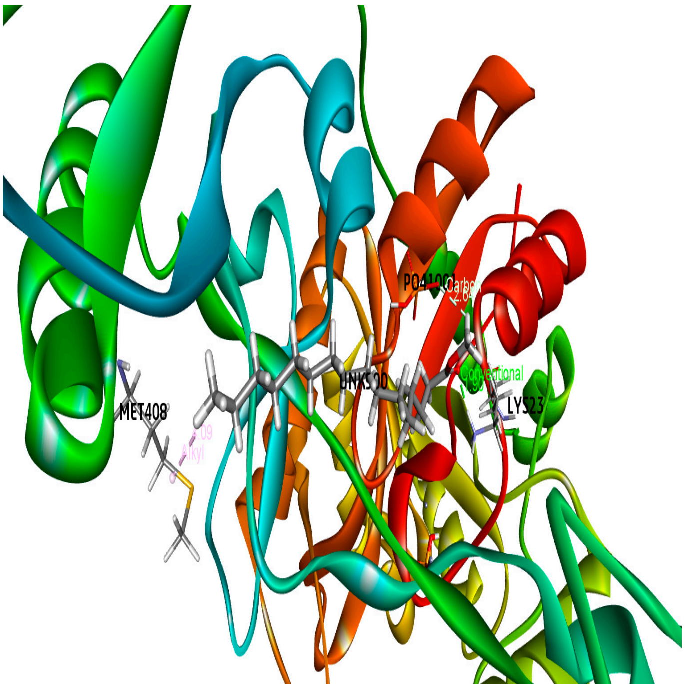
MET408
LYS23

C

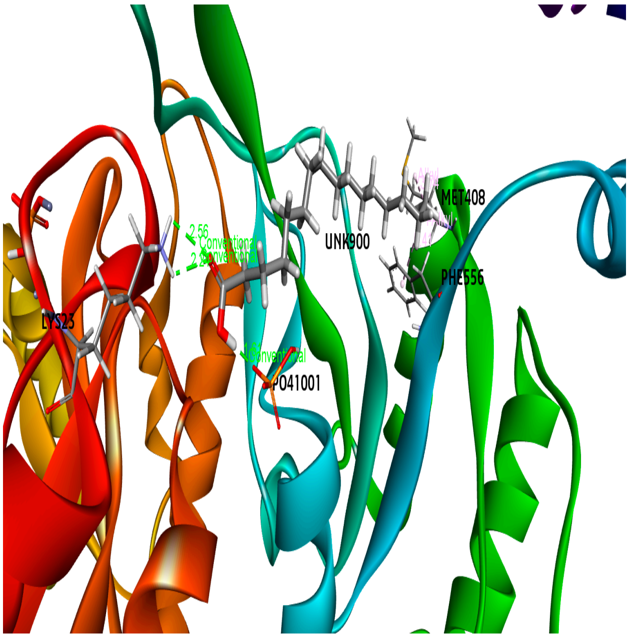
MET408
UNK900
PHE556
LYS23
PO41001

D

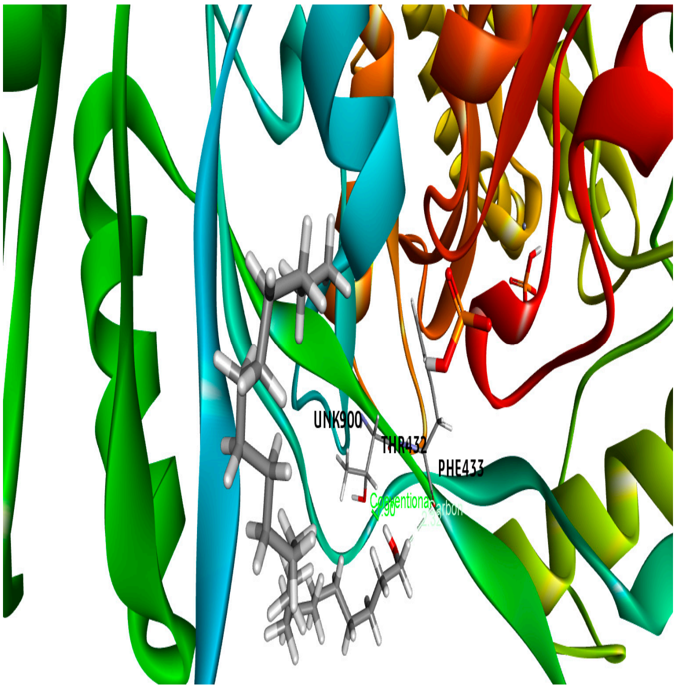
UNK900
THR432
PHE433

E

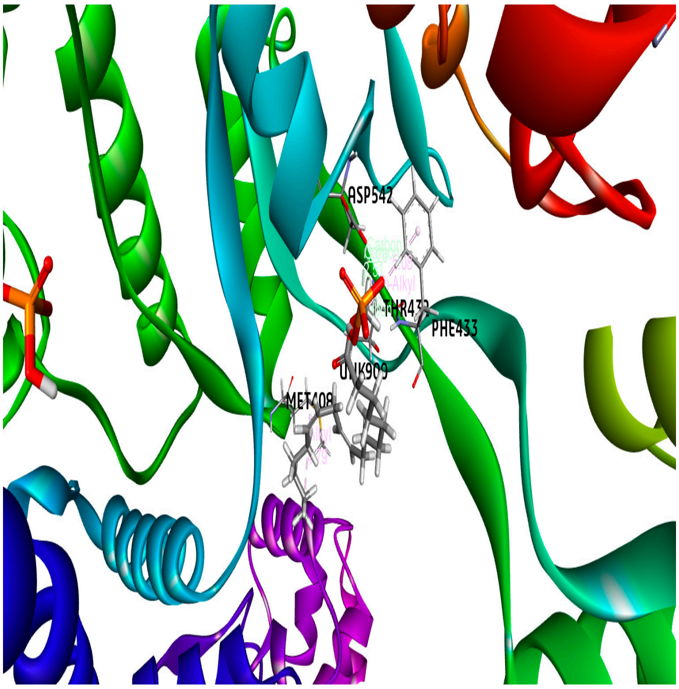
ASP542
PHE433

F

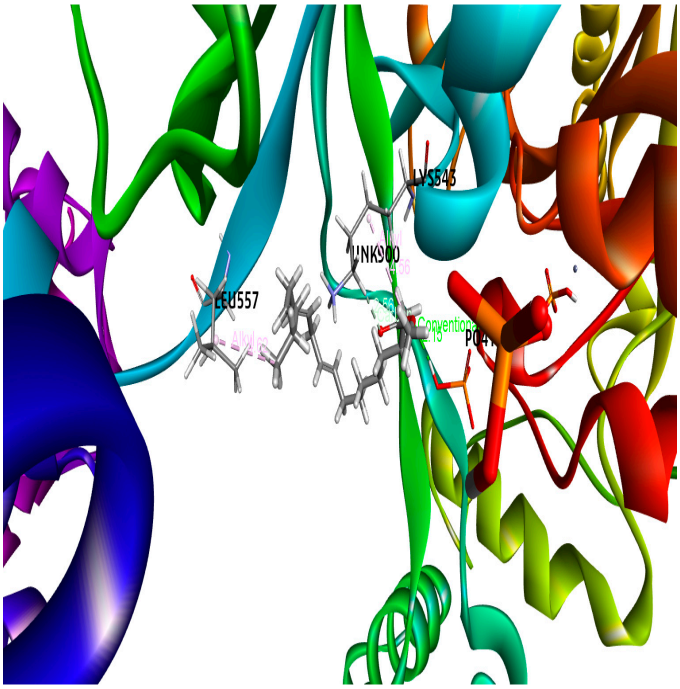
LYS543
UNK900
LEU557

G

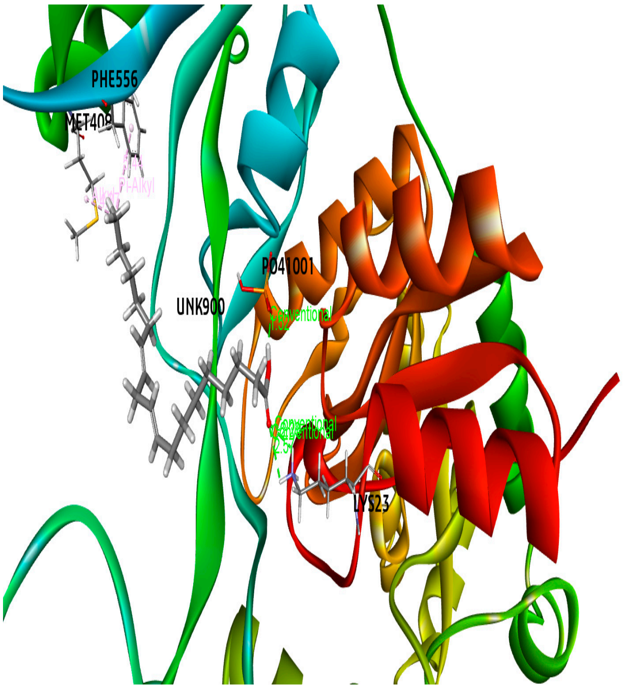
PHE556
UNK900
PO41001
LYS23

H

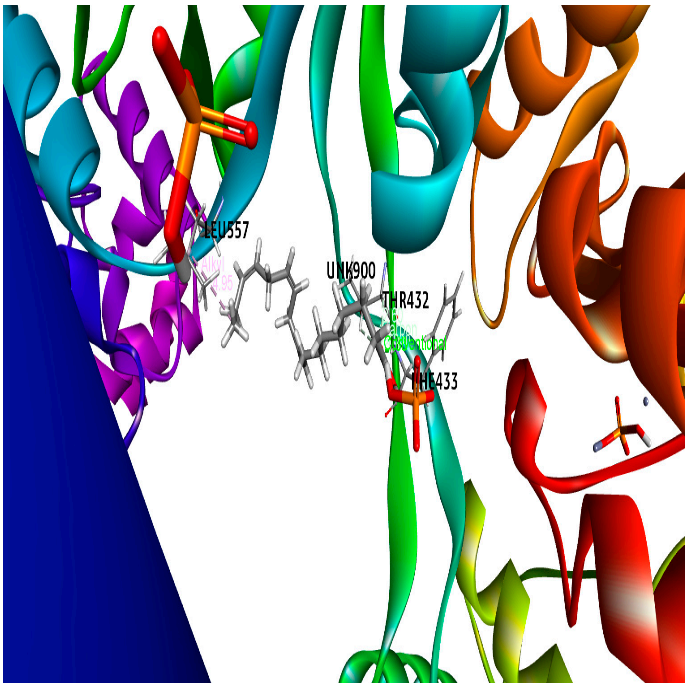

I

Figure 4. 3D Protein-Ligand complex for (A) Pentadecene, (B) Nonadecene, (C) Methyl palmitate, (D) Palmitic acid, (E) Behenic alcohol, (F) Methyl lineoleate, (G) Methyl elaidolinolenate, (H) Acidelinoleique, (I) Linolenic acid docked the DNA polymerase III subunit alpha (PDB ID : 4JOM).

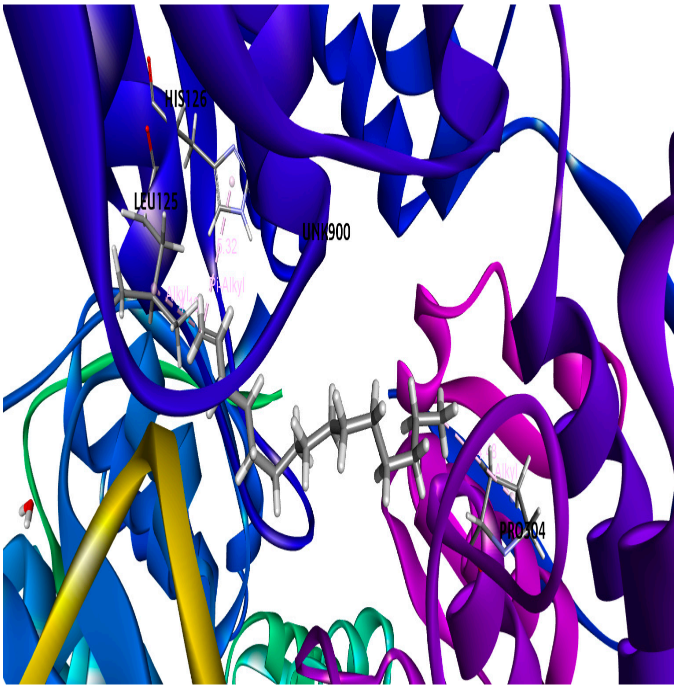
HIS126
LEU125
UNK900
PRO304

A

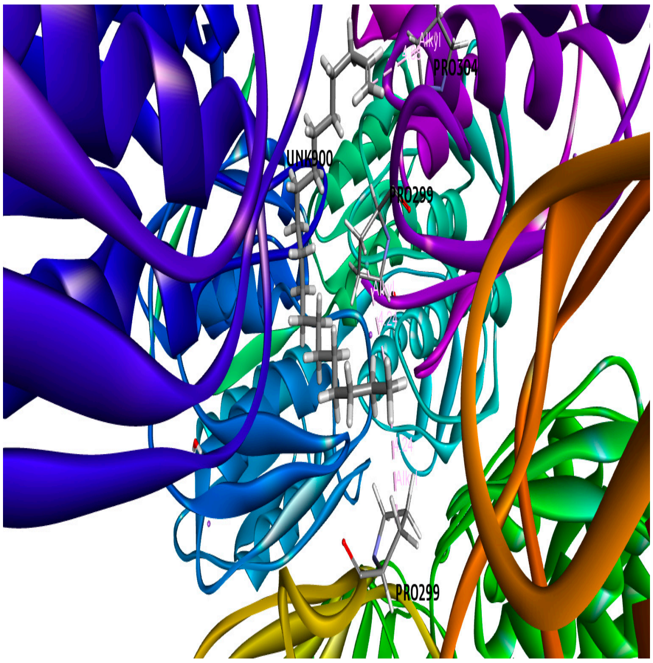
PRO304
UNK900
PRO299
PRO299

B

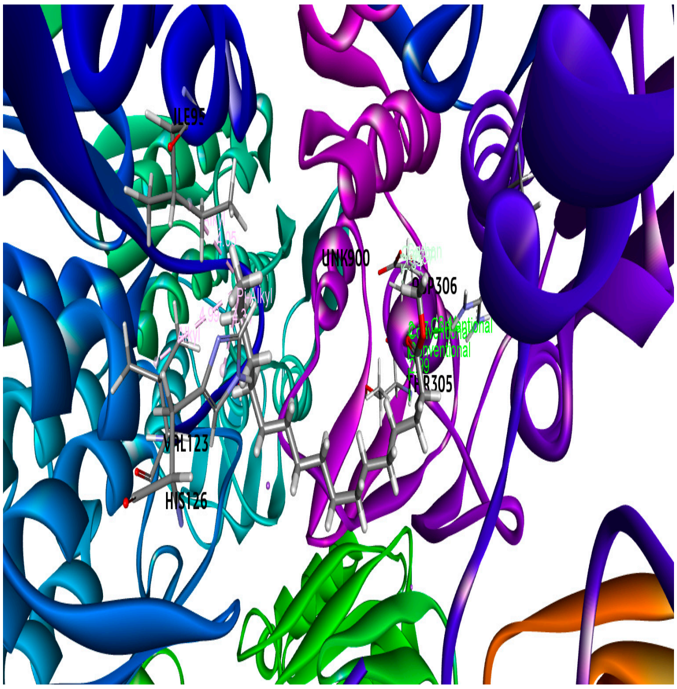
ILE95
UNK900
HIS126

C

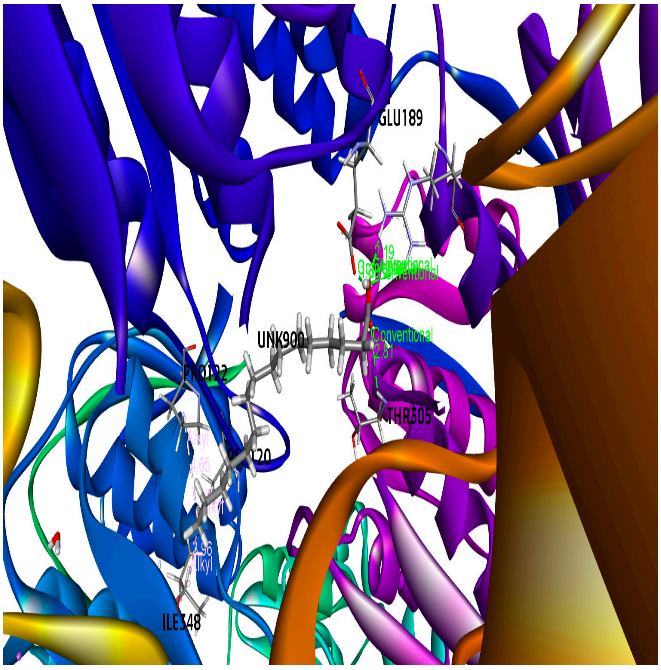
GLU189
UNK900
THR305
ILE348

D

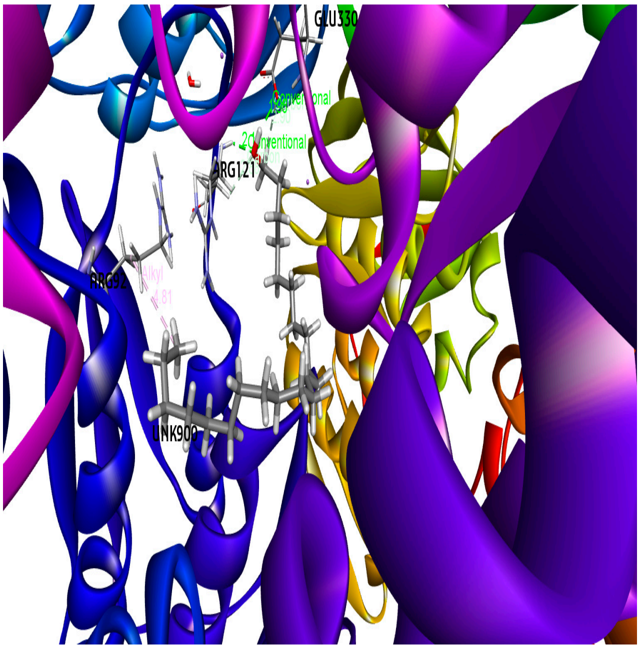
GLU330
ARG121
ARG92
LNK900

E

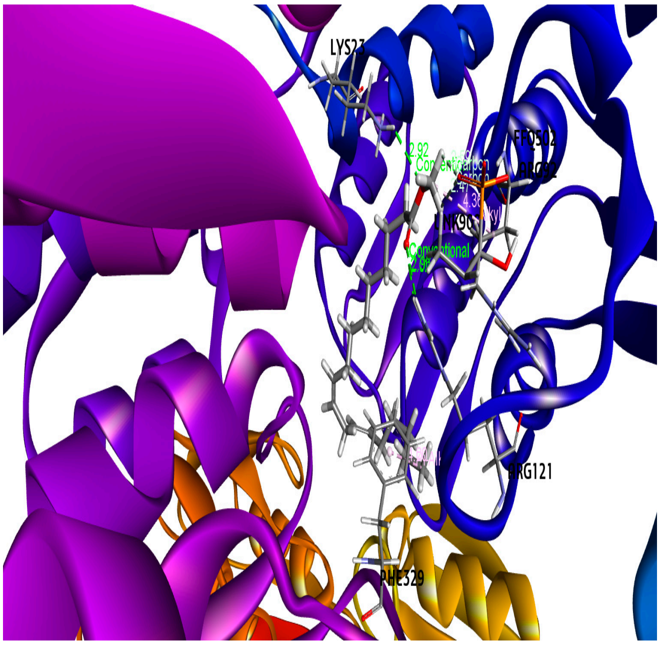
FFQ502
ARG121
PHE329

F

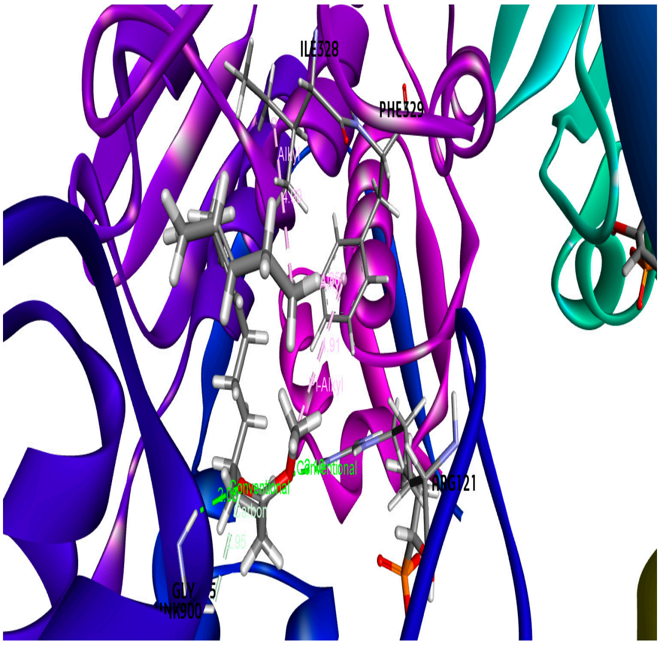
ILE328
PHE329
ARG121
LNK900

G

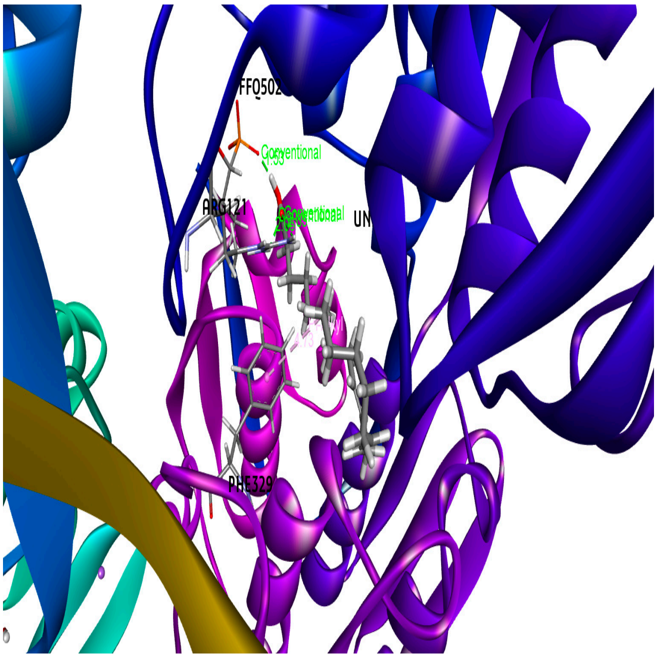
ARG121
PHE329

H

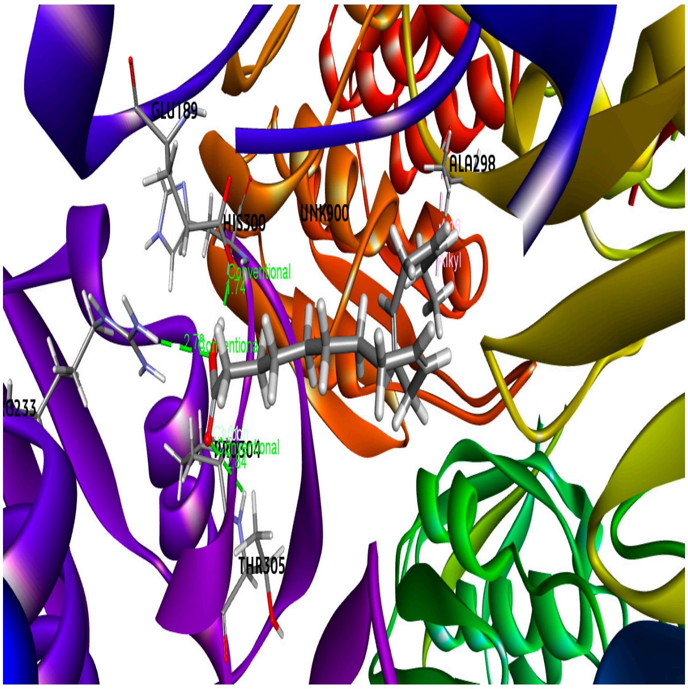

I

Figure 5. 3D Protein-Ligand complex for (A) Pentadecene, (B) Nonadecene, (C) Methyl palmitate, (D) Palmitic acid, (E) Behenic alcohol, (F) Methyl lineoleate, (G) Methyl elaidolinolenate, (H) Acidelinoleique, (I) Linolenic acid docked the UDP-*N*-acetylglucosamine-1-carboxyvinyltransferase (PDB ID : 4R7U).
